# Bicodon bias can determine the role of synonymous SNPs in human diseases

**DOI:** 10.1186/s12864-017-3609-6

**Published:** 2017-03-13

**Authors:** Christina McCarthy, Alejandra Carrea, Luis Diambra

**Affiliations:** 1 Centro Regional de Estudio Génomicos, Universidad Nacional de La Plata, Boulevard 120, La Plata, Argentina; 2 CONICET, Buenos Aires, Argentina; 3 Departamento de Informática y Tecnología, Escuela de Ciencias Agrarias, Naturales y Ambientales, Universidad Nacional del Noroeste de la Provincia de Buenos Aires, Pergamino, Argentina

**Keywords:** Synonymous codon usage, Co-translational folding, Human diseases, Codon pairs, Genetic code

## Abstract

**Background:**

For a long time synonymous single nucleotide polymorphisms were considered as silent mutations. However, nowadays it is well known that they can affect protein conformation and function, leading to altered disease susceptibilities, differential prognosis and/or drug responses, among other clinically relevant genetic traits. This occurs through different mechanisms: by disrupting the splicing signals of precursor mRNAs, affecting regulatory binding-sites of transcription factors and miRNAs, or by modifying the secondary structure of mRNAs.

**Results:**

In this paper we considered 22 human genetic diseases or traits, linked to 35 synonymous single nucleotide polymorphisms in 27 different genes. We performed a local sequence context analysis in terms of the ribosomal pause propensity affected by synonymous single nucleotide polymorphisms. We found that synonymous mutations related to the above mentioned mechanisms presented small pause propensity changes, whereas synonymous mutations that were not related to those mechanisms presented large pause propensity changes. On the other hand, we did not observe large variations in the codon usage of codons associated with these mutations. Furthermore, we showed that the changes in the pause propensity associated with benign sSNPs are significantly lower than the pause propensity changes related to sSNPs associated to diseases.

**Conclusions:**

These results suggest that the genetic diseases or traits related to synonymous mutations with large pause propensity changes, could be the consequence of another mechanism underlying non-silent synonymous mutations. Namely, alternative protein configuration related, in turn, to alterations in the ribosome-mediated translational attenuation program encoded by pairs of consecutive codons, not codons. These findings shed light on the latter mechanism based on the perturbation of the co-translational folding process.

**Electronic supplementary material:**

The online version of this article (doi:10.1186/s12864-017-3609-6) contains supplementary material, which is available to authorized users.

## Background

**Fig. 1:**
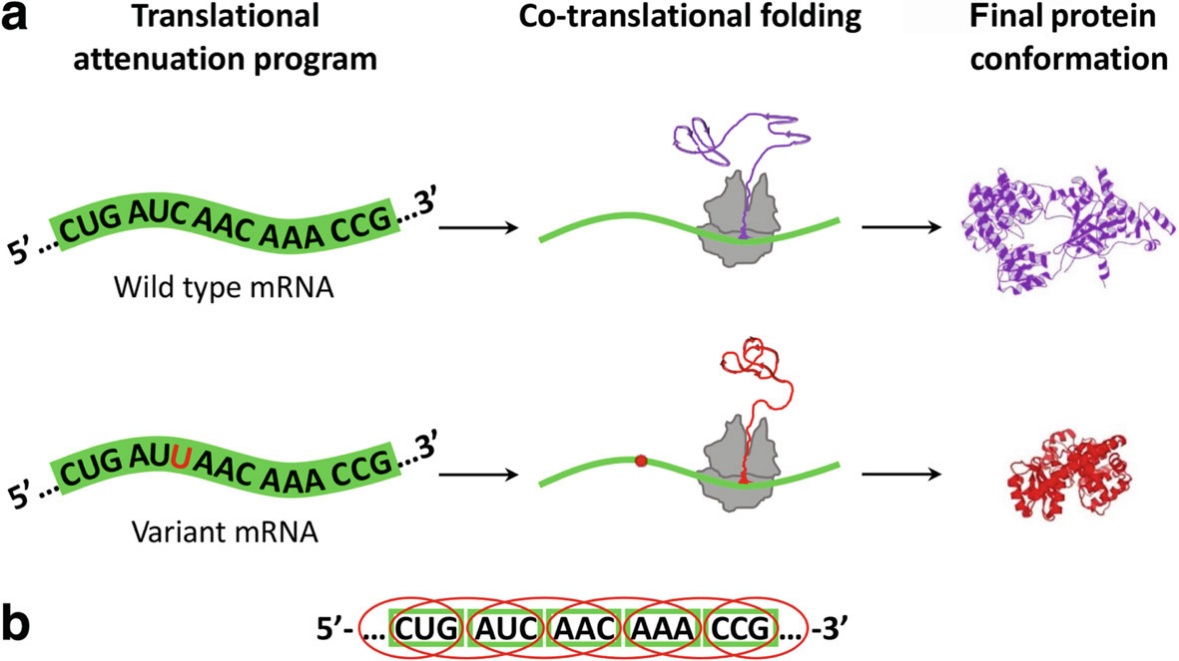
Ribosome-mediated translational attenuation program and bicodons. **a** Graphical representation of how a sSNP can alter the ribosome-mediated translational attenuation program and, ultimately, final protein conformation. Consequently, this can affect protein function leading to pathological phenotypes. **b** Simplified graph showing codons (*green boxes*) and bicodons (*red ellipses*) within an mRNA sequence. Owing to the superposition of bicodons, each codon is part of two different bicodons in a given open reading frame. Alterations of the ribosome-mediated translational attenuation program, with the resulting alternative protein conformations and functions, could be encoded by bicodons, rather than by codons

Recent advances in sequencing and genotyping technologies have allowed the association of pathological traits or diseases with common genetic variants observed in human populations. As a result of genome-wide association studies (GWASs), a large number of human diseases have been associated with single nucleotide polymorphisms (SNPs). These small genetic variants can occur in gene-coding regions or in non-coding regions (introns or intergenic regions). SNPs in a coding region can result in a codon that encodes a different amino acid (missense mutation) or in a premature stop signal (nonsense mutation). Depending on the position and/or the chemical properties of the replaced amino acid, these mutations can lead to nonfunctional proteins, causing human genetic diseases such as epidermolysis bullosa [1], sickle-cell anemia [2], mandibuloacral dysplasia [3], SOD1-mediated amyotrophic lateral sclerosis [4] and cancer [5, 6], among others. Due to the degeneracy of the genetic code (i.e., the existence of more codons than the number of different amino acids and stop signals), a SNP does not necessarily change the amino acid sequence of a translated protein. These types of substitutions are known as synonymous SNPs (sSNPs) and, for a long time, they were considered as silent mutations because it was assumed that they had no phenotypic consequences. However, strong evidence currently supports the fact that synonymous codons have phenotypic consequences, and the notion that sSNPs are innocuous has dramatically changed in the last decade. Synonymous mutations can lead to disease by means of four known mechanisms [7]. One of them is by disrupting splicing signals which, in turn, results in the loss, or gain, of one or more gene-coding regions [8–10]. Another mechanism affects the regulatory binding-sites of transcription factors and miRNAs [11]. For example, Crohn’s disease is caused by synonymous variant rs10065172 within the *IRGM* coding region, which alters a miR-196 binding site [12]. In the third mechanism, synonymous mutations can change the secondary structure of mRNA causing low protein expression levels. An emblematic example of the latter is the sSNP rs769223. This mutation results in a more stable mRNA molecule, but in lower levels of the COMT protein, leading to higher pain sensitivity [13]. The fourth alternative consists in an alteration of the ribosome-mediated translational attenuation program (i.e., a decrease in the translational rate due to ribosomal pauses) which alters protein conformation (Fig. 1a). In this sense, there is strong evidence supporting the fact that synonymous codons are not always translated in the same manner. On the contrary, some of them are translated faster and/or more accurately than others. There are many studies suggesting that ribosomal pauses schedule co-translational folding of protein domains, and determine the final protein conformation [14–24]. This means that sSNPs can alter the ribosome-mediated translational attenuation program with noticeable impact on the final protein configuration and function. This could certainly lead to pathological phenotypes when considering clinically important proteins. In this regard, it has been observed that the drug-transport pump MDR1 protein changes its substrate specificity as a consequence of the sSNP rs1045642 [14], leading to multidrug resistance in cancer cells [25]. Similarly, recently it was proved that the kinetics of co-translational folding (and translation) of the gamma-B crystallin protein is controlled by synonymous codon usage [18].

Despite building evidence which suggests that synonymous codons could be a subliminal code for protein folding [26–29], the operating mechanism for such a secondary code remains poorly understood. To understand how sequences can control the ribosome-mediated translational attenuation program, it is convenient to review the degeneracy of the genetic code. This redundancy offers a lot of degrees of freedom to codify proteins. Nevertheless, organisms use or explore an insignificant fraction of these available possibilities. This fact is a consequence of many biological constraints (such as protein expression levels, ribosomal proofreading errors, protein solubility, folding accuracy, protein stability) operating to optimize how to convey the correct message from genes to functional proteins. As a consequence of these constraints, there are selective pressures that promote a biased usage of synonymous codons [30, 31]. Since the recruitment of a charged tRNA to the codon in the A-site depends on the abundance of each tRNA [32–35], there is a generalized idea that codon usage can modulate translational rates. Another way to modulate the translational rate consists of the non-Watson-Crick (wobble) interactions, since they are usually associated with higher dissociation rates between the mRNA and the decoding center [36]. Nevertheless, transcript translation is a sequential process in which ribosomes synthesize proteins. At each proofreading step the ribosome coordinates several simultaneous or successive tasks, such as the recruitment of a charged tRNA to the codon in the A-site, the assembly of the new residue to the nascent polypeptide, the translocation of the tRNA-mRNA complex, the dissociation of the empty tRNA from the mRNA, and its release from the E-site. Thus, the translational rate is the result of several concomitant processes with different kinetics. In particular, the dissociation process of the mRNA codon from the decoding center has been described as a rate-limiting one [37]. This implies that attenuation of the translational rate not only occurs when the ribosome awaits the entry of scarce tRNA into the A-site, but also during the mRNA translocation process. Consequently, it can be expected that selective pressure operates beyond codon usage. In fact, recent genome-wide statistical analyses have revealed that bicodons, i.e., pairs of consecutive codons, are also subject to evolutionary pressure, and biased bicodon usage has been reported as well [38, 39]. More recently, a genome-wide statistical analysis revealed that some bicodons are overused in sequences associated with highly abundant proteins, but underused when they code for lowly abundant proteins [40]. The opposite situation was also observed, as there are bicodons which are frequently used to codify lowly abundant proteins, but underused in sequences that code for highly abundant ones [40]. Based on the statistical analysis of bicodon usage in sequences associated with lowly or highly abundant proteins, the author estimated a pause propensity measure of all bicodons, and proposed that alternative protein configurations could be associated with alterations in the translational attenuation program encoded by bicodons (Fig. 1 b).

In the light of these previous findings, in this work we reexamined several human genetic diseases associated with sSNPs (compiled by Sauna et al. [41]), by means of computing the relative change in the pause propensity measure due to synonymous bicodon change. We also made the comparison with a similar measure based on the codon frequency usage, and found that in many cases the diseases of Sauna’s list can be better explained by an alteration of the ribosome-mediated translational attenuation program encoded by bicodons rather than by codons. Finally, we showed that the changes in the pause propensity of the sSNPs related to diseases are significantly greater than the pause propensity changes associated with clinically benign sSNPs.

## Results

### Bicodon bias in the human genome

**Fig. 2:**
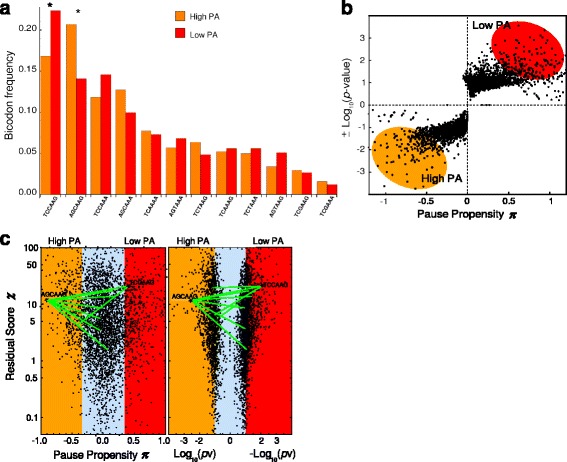
Relationship between synonymous bicodons, protein abundances (PA) and pause propensity. **a** Frequency distributions associated with the bicodons that encode the amino acid pair SK, computed using sequences from the low PA sample (*red bars*), and from the high PA sample (*orange bars*). Some bicodons are more frequent within lowly abundant proteins (such as TCCAAG), some within highly abundant ones (such as AGCAAG), and other bicodons have similar frequencies in both groups of proteins (such as TCAAAA, TCAAAG and TCGAAG). **b** Raster plot of the *p*-values *versus* the pause propensity for all bicodons. To improve the visualization of this correlation we plot −*S* log10[*p*-value] instead of *p*-value, where *S* takes the values +1 or −1 when the bicodon has preference for sequences with low or with high PA, respectively. **c** Raster plot of the residual scores *χ* ^2^ *versus* the pause propensity and *p*-values for all bicodons. Small pause propensity values (*orange zone*) are related to bicodons with high PA, whereas large pause propensity values (*red zone*) are related to bicodons with low PA. *Green lines* represent the nine synonymous bicodon variants for the SK amino acid pair that involve a large change in pause propensity (*Δ* *π*≥0.754)

Since we proposed that synonymous mutations programmed by bicodons can result in alterations in the timing of co-translational folding (which in turn can lead to pathological phenotypes), in this work we focused on the mechanism by which translational rate, and consequently co-translational folding too, are altered due to ribosomal pauses. In a previous article we related bicodon sequences to their pause propensities, by means of counting bicodon occurrence in coding sequences associated with highly or lowly abundant proteins. For illustrative purposes, Fig. 2 a depicts the frequency distributions associated with the bicodons that encode the amino acid pair SK. These distributions were computed using sequences from the low protein abundance (PA) sample (red bars), and from the high PA sample (orange bars). While bicodons TCAAAA, TCAAAG and TCGAAG have similar frequency usage in both sequence samples (low and high PA), many other bicodons have an evident preference for sequences related to low or high PA. In particular, the bicodons TCCAAG and AGCAAG have a high preference for coding proteins associated with low and high abundance, respectively. As shown in [40], the observed bias in bicodons cannot be explained by codon usage. This leads to questions concerning the selective forces that drive this bicodon bias.

Considering that sequences which codify highly abundant proteins need to be optimized in terms of translational rate, we have interpreted this bias by assigning a key role to bicodons in programming translation pauses of the ribosomal machinery. Using the occurrence frequency in each sequence sample, we have computed the pause propensity measure *π* for each bicodon, and applied the Fisher’s exact test to asses the statistical significance of the usage bias. In addition, we have also computed the residual score *χ* ^2^, defined in [42], which indicates when the observed bicodon bias can be explained, or not, by the codon usage bias. Notice that while *π* expresses a degree of the preference of a given bicodon for coding low PA sequences instead of high PA sequences, the *p*-value provided by Fisher’s exact test is the probability against the null hypothesis of equal distribution in both sequence samples. The latter can be more influenced by the number of observations rather than by the net difference of bicodon occurrence between samples. Figure 2 b depicts a raster plot between *π* and the *p*-value. Note that, for a better visualization, the signed log[*p*-value] has been plotted.

This figure also shows that a high preference (i.e. high |*π*|), not necessarily implies a low *p*-value. However, some bicodons present a significant preference for coding low (red balloon) or high PA sequences (orange balloon).

**Fig. 3:**
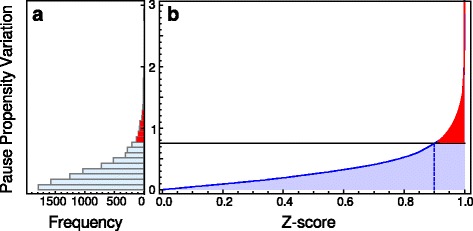
Pause propensity variation and *Z*-score. **a** Histogram of the pause propensity variation of synonymous bicodon variants associated with sSNPs. **b** Associated *Z*-score. The *red* region indicates the highest 10% pause propensity variation, i.e., those sSNPs with a pause propensity variation larger than *Δ* *π*=0.754

Figure 2 c depicts two raster plots of the residual scores *χ* ^2^ *versus* the pause propensity and the *p*-values for all bicodons. Large values of the residual score (*χ* ^2^>5) indicate that the observed bias in the bicodon cannot be explained by codon usage [42]. Our working hypothesis is that bicodons with high values of the pause propensity measure, such as bicodon TCCAAG, could be linked to ribosomal pauses (red zone in Fig. 2 c). A synonymous bicodon variant consists of a change in one or more nucleotides of the hexanucleotide, that still codifies for the same amino acid pair. When one of these variants changes the associated pause propensities, it can alter the translational attenuation program leading to protein misfolding and dysfunction. In the case of our particular example, the amino acid pair SK can be codified by 12 synonymous bicodons, allowing 66 alternative synonymous bicodon variants. Nine of such synonymous variants (green lines in Fig. 2 c) involve a relative large change in the pause propensity (*Δ* *π*≥0.754). Among these, there is only one that corresponds to a single point mutation: the mutation AG**T**AAG ⇔ AG**C**AAG (with *π*= 0.14 and *π*= -0.90, respectively, which represents a change of *Δ* *π*=1.04). These bicodons have an associated *p*-value of 0.065 and 0.003, respectively, which means that the preference of bicodon AG**C**AAG for coding highly abundant proteins is significant. On the other hand, none of the synonymous bicodon variants for the KS amino acid pair involve a large change in the pause propensity. Codon usage bias *per se* cannot explain the lack of symmetry between amino acid pairs SK and KS, as well as with many other pairs, because both pairs have the same codons but in a different order. An analysis of all possible 420 amino acid pairs (including residue:stop) results in that there are 26718 synonymous bicodon variants, 8497 of which correspond to sSNPs. Figure 3 a displays a histogram of the pause propensity variation of all possible synonymous bicodon variants associated with the sSNPs. Figure 3 b depicts the associated *Z*-score, or quantile function, where the red region indicates the highest 10*%* pause propensity variation, i.e., those sSNPs with a pause propensity variation larger than *Δ* *π*=0.754. A similar *Z*-score which was calculated for the measure based on the codon frequency usage is depicted in the Additional file 1: Figure S1.

### Analysis of pathological and benign sSNPs in humans

Sauna et al. compiled a list of synonymous mutations in human genes that significantly correlate with 22 human diseases [41]. This compilation only includes those sSNPs that have a significant association with disease or a clinical trait. One of these, the multidrug resistance case, has been confirmed to be associated to changes in protein conformation due to alterations in the translational attenuation program [14]. It is also known that the underlying mechanisms of Crohn’s disease, cystic fibrosis, and temporomandibular joint disorder (TMD) are not related to alterations of the translational attenuation program. On the other hand, in the remaining 18 diseases the reason why the sSNPS lead to pathological conditions remains unknown. In this paper, we analyzed the local sequence context of each compiled sSNP, by computing the pause propensity change due to the sSNP in both bicodons affected by the variation. The results of this analysis are summarized in Table 1, and further details of our analysis for each disease are given in Additional file 2: Text and Additional file 3: Table S1.

**Table 1:** Pause propensity variation due to sSNPs linked to clinically relevant genetic diseases or traits

Disease/trait	Gene	rsID	Bicondon change		*Z*-score	
			from	to	bicodon	codon
Macular degeneration	CFHR5	rs34533956	GA**C**GTG	GA**T**GTG	0.91	0.51
Longevity	TERT	rs33954691	CA**C**GCA	CA**T**GCA	0.84	0.71
		rs33959226	GC**A**GAG	GC**G**GAG	0.83	0.55
Asthma	SLC6A7	rs2240794	GA**T**AGC	GA**C**AGC	0.11	0.51
Pul. sarcoidosis	CARD15	rs1861759	GTGCG**T**	GTGCG**G**	0.99	0.17
Tuberculosis	TIRAP	rs7932766	GC**C**TAC	GC**T**TAC	0.74	0.62
Cystic Fibrosis	CFTR	rs1800092	AT**C**ATC	AT**A**ATC	0.09	0.17
Coeliac disease	CD44	rs1071695	CA**C**GTG	CA**T**GTG	0.84	0.71
	APIP	rs1571133	AC**A**CTT	AC**C**CTT	0.32	0.39
Crohn’ disease	IRGM	rs10065172	CTGATG	**T**TGATG	0.77	0.93
Smoking-related cancer	NBS1	rs709816	GA**T**GCA	GA**C**GCA	0.80	0.51
		rs1061302	AATCC**A**	AATCC**G**	0.79	0.47
Colorectal cancer	ERCC1	rs11615	AA**T**GTG	AA**C**GTG	0.97	0.07
Chronic myeloid leukemia	WT1	rs2229069	CGCAC**G**	CGCAC**A**	0.80	0.24
		rs2227985	CAGGA**A**	CAGGA**G**	0.69	0.10
Non-small-cell lung carcinoma	EGFR	rs2293347	ACAGA**C**	ACAGA**T**	0.48	0.51
Cervical & vulvar cancer	IL2	rs2069763	CT**G**CTG	CT**T**CTG	0.49	0.80
Drug resistance	ABCB1	rs1045642	AT**T**GTG	AT**C**GTG	0.94	0.55
	CHRNA4	rs1044396	CCGAG**C**	CCGAG**T**	0.26	0.74
Alzheimer	COX6B1	rs7991	AAGAC**C**	AAGAC**T**	0.84	0.36
	COX6C	rs1130569	TA**C**GAT	TA**T**GAT	0.59	0.24
	COX8A	rs61759492	AT**C**ATG	AT**A**ATG	0.83	0.17
ADHD	NTF3	rs6332	CAGCC**G**	CAGCC**A**	0.46	0.47
Huntington	ADORA2A	rs5751876	GGCTA**T**	GGCTA**C**	0.99	0.24
	PADI2	rs2076615	GG**T**GGC	GG**G**GGC	0.93	0.77
Schizophrenia	SYNGR1	rs74681509	ACCTT**C**	ACCTT**T**	0.84	0.36
	DRD2	rs6277	ACTCC**C**	ACTCC**T**	0.95	0.82
		rs6275	CACCA**T**	CACCA**C**	0.81	0.71
TMD	COMT	rs769223 ^∗^	GC**G**AGG	GC**A**AGG	0.67	0.55
		rs1121923	GT**G**GCC	GT**A**GCC	0.89	0.13
Type III		rs248	GA**G**TTT	GA**A**TTT	0.34	0.10
hyperlipidemia	LPL	rs45607438	CA**T**GTT	CA**C**GTT	0.89	0.71
		rs316	AAGAC**C**	AAGAC**A**	0.91	0.39
Chronic hepatitis C	IRF7	rs1061501	CG**G**GAT	CG**A**GAT	0.84	0.17
Osteoporosis	CD44	rs11033026	CATGA**G**	CATGA**A**	0.93	0.10

We computed the *Z*-score associated to the change in the pause propensity values, or to the change in the differential RSCU, as a consequence of single nucleotide mutations in a subset of 22 human diseases or traits selected from [41]. The first column lists the diseases/traits, the following columns list the affected genes, the rs IDs, the bicodon changes, and *Z*-scores. Only the variant associated with larger *Z*-score is listed, for a more complete table, please see Additional file 3: Table S1. ^∗^ is validated by HapMap

We found that the variations of the pause propensity measure associated with the corresponding sSNPs, are low in the case of the following diseases: Crohn’s disease, cystic fibrosis and TMD; whereas it is high in the multidrug resistance case, in perfect agreement with the known mechanisms of these diseases. Performing the same analysis for the remaining 18 diseases with unknown subjacent mechanisms, we found that 14 of these have large associated changes (*Z*-score ≥0.80) in the pause propensity due to the sSNPs in one or more bicodon variants, in at least one of the genes implicated in the disease. We also observed that the affected bicodons are associated with a significant usage bias, as determined by Fisher’s exact test. For comparison we also computed a similar measure based on codon frequencies, the differential relative synonymous codon usage (RSCU). In contrast with previous observations, the differential RSCU only shows large changes in the case of cervical and vulvar cancer (rs2069763), schizophrenia (rs6277), in agreement with the bicodon analysis, and Crohn’s disease (rs10065172), where a large change in the pause propensity is not expected because, in this case, the sSNP is related to alterations in the miR-196 binding site [12]. Furthermore, the *p*-value provided by Fisher’s exact test associated with codons does not vary significantly (Additional file 3: Table S1). These results add suggestive evidence supporting the hypothesis that sSNPs that do not alter splicing processes neither mRNA structure, but are associated with diseases, could be involved in the alteration of the ribosome-mediated translational attenuation program. Moreover, this attenuation program could be more related to bicodons than to codons. On the other hand, we did not observe large changes in the pause propensity measure for the remaining four diseases, which could be indicating that they are not associated with alterations of the ribosome-mediated translational attenuation program. However, we found that in two of them, asthma and cervical and vulvar cancer, the corresponding sSNPs introduce rare bicodons in place of more frequent ones. In particular, in the case of asthma (rs2069763), the frequency usage of the GA**T**AGC bicodon over both samples sequences, is 6-fold smaller than GA**C**AGC, while in the case of cancer (rs2069763), the frequency usage of the CT**G**CTG bicodon is 4-fold higher than the frequency usage of CT**T**CTG. As these bicodons are not frequent in both the low and high PA samples, we are not able to relate this rareness with the ribosome-mediated translational attenuation program, or with other known mechanisms that lead to a pathological condition. However, it would seem that bicodon frequency could be an indicator of the sSNP pathogenicity. Finally, in the case of non-small-cell lung cancer and attention-deficit/hyperactivity disorder (ADHD), our local sequence context analysis did not show alterations associated with these sSNPs.

To further analyze synonymous mutations which correlate with human diseases, we also performed our local sequence context analysis for sSNPs which have been classified clinically as benign, as a control test. The results of this analysis are summarized in Additional file 4: Table S2. These results indicate that benign sSNPs have an associated lower change in the pause propensity measure, compared to sSNPs which we have associated to alterations of the ribosome-mediated translational attenuation program. In this sense, we used the Mann-Whitney *U* test to analyze the difference in pause propensities between pathological and benign sSNP groups. This test identified significant differences between both groups (*p*-value =0.0022). In contrast, a similar analysis with the differential codon usage did not identify significant differences between both groups (*p*-value =0.41).

Even though we found significant differences in the pause propensities values between pathological and benign sSNP groups, some benign sSNPs (like rs11797, rs2242244, and rs2272068, among others) have large alterations of their pause propensities (see Additional file 4: Table S2). This could mean that a large value of *Δ* *π* might be a necessary condition for an alteration of the translational attenuation program. However, this is not a sufficient condition to associate a sSNP with a disease.

## Discussion

Genetic diversity allows populations to adapt to environmental changes, making them less prone to extinction. From a clinical point of view, such genetic variability also underlies the distinctive susceptibility of organisms to diseases and their differential sensitivity to toxins or drugs. SNPs are the most common form of such diversity, and account for much of the variation in genetic traits between individuals. It was originally thought that sSNPs had no implications on human health. Nevertheless, this concept has radically changed in the last ten years, since it was shown that the supposed ’silent’ mutations can have an impact on human health through various mechanisms. One of these mechanisms is protein misfolding, which occurs by means of an alteration of the ribosome-mediated translational attenuation program [15]. It was demonstrated that this mechanism operates in the misfolding of membrane protein Pgp encoded by the *ABCB1* gene [14], in conformational changes in the FRQ protein of *Neurospora* [43], in the SufI protein of *E. coli* [17], and in the bovine gamma-B crystallin [18]. However, it is not known how widespread this mechanism is among the ensemble of clinically relevant genetic traits or diseases.

The main idea underlying the protein-misfolding based mechanism, is that codon usage can modulate ribosome traffic and, consequently, co-translational folding, by means of associating frequent codons with fast elongation rates and rare codons with slow elongation rates. However, the exact mechanism connecting proteostasis with codon usage remains unclear. Recently it was suggested that ribosome translocation from the codon in the P-site to the codon in the A-site depends on both codons, and not only on the codon in the A-site [40]. Consequently, the ribosome-mediated translational attenuation program would be encoded by bicodons, rather than by single codons. The author defined a pause propensity measure based on the preference of each bicodon to encode sequences associated with highly abundant or lowly abundant proteins.

In the light of this new concept, in this article we reviewed 22 genetic diseases or traits associated with synonymous mutations. It is known that the genetic condition for multidrug resistance is a consequence of the protein-misfolding based mechanism [14], whereas three other of these 22 diseases (Crohn’s disease, cystic fibrosis and TMD) are the result of different mechanisms. Nevertheless, the underlying mechanisms of the remaining diseases or traits are still unknown.

## Conclusions

Our results, based on the variation of the pause propensities associated with the corresponding sSNPs, are in agreement with the underlying mechanism of the four diseases with known causes. Regarding the other 18 diseases, we have found that 14 of them could be explained by one or more alterations in the translational attenuation program in at least one of the genes implicated in the disease. These alterations, which are results of the sSNPs, consist in large changes in the pause propensity associated with bicodons. We have found that these changes are significantly greater than those related to clinically benign sSNPs. However, a large change in the pause propensity does not necessarily lead to a misfolded protein, even less so to a pathological condition. A pathological condition will be determined by how the function of the protein is affected, and by the role of the pause on protein folding, among others. On the other hand, we have found that differential RSCU has similar values in both pathological and benign sSNP groups. This suggests that synonymous codons could have a secondary role in determining translational pauses, in any case derived from the bicodons that they form.

We believe that the findings presented here, even though preliminary, shed light on genetic diseases associated with sSNPs whose underlying mechanism is based on protein misfolding due to a modification of the translational attenuation program encoded in bicodons. Future experimental studies will test our predictions helping to understand the role of bicodon preferences in proteostasis in a more conclusive manner.

## Methods

The methodology that was used in this work comprised two steps: (i) the computation of the *p*-value provided by the Fisher’s exact test, the residual score and the pause propensity for all bicodons from human coding sequences; and (ii) the computation of the pause propensity variation, and the associated *Z*-score function, due to the sSNPs linked to each human genetic disease considered here. In our study, we have considered a list of genetic human diseases linked to sSNPs compiled by Sauna et al. [41]. This list comprises 50 sSNPs that have significant association with diseases, but does not include sSNPs with literature reports that identified both synonymous and non-synonymous mutations. From this list, we only considered those SNPs which are validated by 1000Genomes, and also rs769223 which is validated by HapMap. This excluded 15 sSNPs from our study, yielding 35 sSNPs in 27 genes related to 22 clinically relevant genetic traits. Additional details of the sSNPs listed in Table 1 can be found in Additional file 3: Table S1. Futhermore, we also selected all those sSNPs from single nucleotide polymorphism database (dbSNP) [44], which are clinically classified as benign, have been validated by 1000Genomes project, and have a minor allele frequency (MAF) greater than 0.05. We found 87 sSNPs following these criteria. Further details are listed in Additional file 4: Table S2.

### Computation of Fisher’s exact test, residual score and pause propensity

The first step for this computation was previously developed in [40]. Briefly, the computation consisted of the frequency count of each bicodon in two samples of coding sequences, one sample associated with lowly abundant proteins and the other sample associated with highly abundant proteins. The sequences constituting each sample were selected according to their abundance, based on the genome-wide protein abundance database (Paxdb) [45, 46]. The nucleotide coding sequences corresponding to the selected proteins were downloaded from the Ensembl web site [47]. Next, we computed bicodon occurrences $o_{ij}^{X}$ over all coding sequences belonging to a given sample *X*, where index *i* denotes the codon corresponding to the P-site, while *j* denotes the one corresponding to the A-site. After this, we used Fisher’s exact test to examine whether the number of occurrences of bicodons, $o_{ij}^{L}$, observed in the sequence sample associated with low PA, was significantly different than the number of occurrences observed in the high PA sample $o_{ij}^{H}$. The *p*-values of all bicodons are depicted in heat map fashion in Additional file 5: Figure S2. To improve visualization, colors are related to the signed log[*p*-value], i.e., the quantity −*S* log10[*p*-value], where *S* takes the values +1 or −1 depending on the preference of the bicodon for low or high PA sequences, respectively.

We also computed the residual score for each bicodon by removing the contribution due to the bias in codons and amino acids. The residual score *χ* ^2^ is given by [42]:

1 $$ \chi_{ij}^{2}= \frac{\left(o_{ij} - {\hat e}_{ij}\right)^{2}}{{\hat e}_{ij}},  $$

where ${\hat e}_{ij}= e_{ij}\times \frac {\sum ^{*}_{kl} o_{kl}}{\sum ^{*}_{kl} e_{kl}} $, (the ^∗^ indicates that the sum is only over bicodons that encode the same amino acid pair encoded by the bicodon *ij*). *e*_*ij*_ is the expected number of occurrences of each bicodon given by $e_{ij}= f_{i} f_{j} N_{p}/N_{tot}^{2}$, where *f*_*i*_ is the number of occurrence of single codons *i*, *N*_*tot*_ is the total number of codons in the set of sequences, and *N*_*p*_ is the number of bicodons. We compute the residual score of each bicodon over low and high PA sample sequences separately, and will be denoted by $\chi ^{2}_{L}$ and $\chi ^{2}_{H}$, respectively. Thus, we use the total residual score $\chi _{ij}^{2}=\chi ^{2}_{L} +\chi ^{2}_{H}$ to assess whether the usage bias in bicodon *ij* can explained by usage bias in codons *i* and *j*.

Besides *p*-values provided by Fisher’s exact test and residual scores *χ* ^2^, we also defined a pause propensity measure *π* that takes the differential frequency usage of bicodons in each sample sequences into account. This measure can be understood in terms of the RSCU index introduced by [48]. RSCU index is defined as RSCU_*i*_=*s* *f*_*i*_/*N*_*a*_. Where *s* is the number of codons encoding the same amino acid, *N*_*a*_ is the frequency of that amino acid, and *f*_*i*_ is the frequency of the codon *i* within the human genome. When RSCU_*i*_ is greater, or lower, than 1, this indicates that codon *i* is over-, or under-, represented in the genome, respectively. Thus, we defined the pause propensity measure *π* as the difference between the relative synonymous bicodon usage computed over the sequence sample associated with low PA, (RSBU ^*L*^), and the one computed over the sequence sample associated with high PA, (RSBU ^*H*^). Mathematically,

2 $$ \pi_{ij}= \text{RSBU}_{ij}^{L} -\text{RSBU}_{ij}^{H} = q \left(f_{ij}^{L} - f_{ij}^{H}\right)/ N_{ap}.  $$

Here, $f_{ij}^{X}$ is the frequency of the bicodon *ij* computed over the sequence sample *X*, *q* is the number of bicodons encoding the same amino acid pair, and *N*_*ap*_ is the frequency of that amino acid pair within the human genome. Thus, a large, or small, value of *π*_*ij*_ indicates the preference of bicodon *ij* for coding low, or high, PA sequences, respectively. The values of *π*, *p*-value, and *χ* ^2^ for all bicodons are listed in Additional file 6: Table S3.

For the sake of comparison, we computed a similar measure to *π* but now in terms of codon frequency. We denoted it as differential RSCU $\left (\text {i.e.,~} \text {RSCU}_{ij}^{L} -\text {RSCU}_{ij}^{H}\right)$. We also performed Fisher’s exact test for codons. The latter allowed us to examine whether the number of occurrences of codons observed in the low PA sample, was significantly different to the number of occurrences observed in the high PA sample.

In our study we have not considered triplets of codons. We believe that the codon associated with the E-site may have low or none effect on ribosomal transit, because this site is not involved in the translocation of the tRNA-mRNA complex. Furthermore, if triplets were to be considered it would be necessary to increase the number of sequences in our samples to a prohibitive size, which would result in poor statistical measures.

### Pause propensity variation of genetic human diseases

There are two bicodon variations associated with one SNP. This occurs because as each SNP takes part in one codon, it can be part of two different bicodons, as indicated in Fig. 1 b. For each variation, bicodon _*i*_→ bicodon _*f*_, we computed the pause propensity variation *Δ* *π*=*π*_*f*_−*π*_*i*_. To assess the statistical significance of this variation we computed a *Z*-score, also known as the inverse cumulative distribution function. For each probability *p* in the probability distribution of a random variable *X*, this score function assigns value *x* for which Prob (*X*≤*x*)=*p* [49]. To this end, we computed *Δ* *π* for 8497 synonymous bicodon variants associated to single point mutations. Figure 3 depicts the histogram of *Δ* *π*, where the right panel depicts the associated *Z*-score. The red region indicates the highest 10*%* pause propensity variation, i.e., those sSNPs with a pause propensity variation larger than *Δ* *π*=0.754. A similar *Z*-score function was computed for the differential RSCU obtained for codons. Additional file 1: Figure S1, depicts the associated *Z*-score function for codons. The Z-score values for bicodons and codons associated with the studied sSNPS are listed in the last column of Tables S1 and S2. These tables also list the relative change of the *p*-value defined as the ratio (*p*v _*f*_−*pv*_*i*_)/*p*v _*i*_, where *p*v _*i*_ and *p*v _*f*_ are the signed log [*p*-value] of bicodon *i* and bicodon *f*, respectively.

## Additional files

Additional file 1
**Figure S1.** *Z*-score function associated with the differential RSCU change of all the synonymous codon variants. The red region indicates the highest 10% pause propensity variation, i.e., those sSNPs with a variation larger than 0.28. (PDF 13 kb)

Additional file 2 Text. Additional details for each disease or clinical trait listed in Table 1, such as basic background, references, accession number and local sequence context analysis. (PDF 88 kb)

Additional file 3
**Table S1.** Genetic diseases and sSNPs. The first column corresponds to the disease or genetic trait. The second and third columns correspond to the associated gene and SNP (where both can be more than one), respectively. Each SNP affects only one codon (third SNP row), but two bicodons, when the mutation is in the P-site (first SNP row), and when the mutation is in the A-site (second SNP row). Fourth, fifth, sixth and seventh columns, refer to the starting bicodon, list the bicodon sequence, the *p*-value, the residual score ($\chi ^{2}=\chi _{L}^{2}+ \chi _{H}^{2}$) and *π* value, respectively. The following four columns, refer to the resulting bicodon, list the bicodon sequence, the log-transformed *p*-value, the residual score and *π* value, respectively. The following three columns enumerate the relative *p*-value, the pause propensity variation, *Δ* *π*, and the corresponding *Z*-score due to the synonymous bicodon variant. The last two columns contain a summarized result of our analysis for each disease and the related references. (XLS 41 kb)

Additional file 4
**Table S2.** Silent sSNPs. First and second columns correspond to benign sSNPs and their associated genes respectively. Each SNP affects only one codon (third SNP row), but two bicodons, when the mutation is in the P-site (first SNP row), and when the mutation is in the A-site (second SNP row), but only one codon (third SNP row). Third, fourth, fifth and sixth columns, refer to the starting bicodon, list the bicodon sequence, the *p*-value, the total residual score ($\chi ^{2}=\chi _{L}^{2}+ \chi _{H}^{2}$) and *π* value, respectively. The following four columns, refer to the resulting bicodon, list the bicodon sequence, the log-transformed *p*-value, the *χ* ^2^ score value and *π* value, respectively. The last three columns enumerate the relative *p*-value, the pause propensity variation, *Δ* *π*, and the corresponding *Z*-score due to the synonymous bicodon variant. (XLS 66 kb)

Additional file 5
**Figure S2.** Pause propensity heat map. The color of each cell is determined by the pause propensity *π* of the associated bicodons. Columns represent codons corresponding to P-site, while rows represent codons corresponding to A-site, so that each cell in the heat map represents a bicodon. Red cells indicate bicodons with the highest pause propensity value (low PA preference), while blue cells indicate bicodons with the lowest pause propensity value (high PA preference). Rows and columns have been clustered to improve visualization. (PDF 260 kb)

Additional file 6
**Table S3.** Statistical features of 3904 bicodons in the human genome. The first column corresponds to the amino acid pair (^∗^ symbolizes stop codon), the second column to the corresponding bicodon, the third and fourth columns list the number of occurrences of bicodons in the low and high PA samples, respectively. The fifth and sixth columns correspond to the residual scores $\chi _{L}^{2}$ and $\chi _{H}^{2}$ computed over the low and high PA samples, respectively. The last two columns correspond to the signed log[*p*-value] and the pause propensity values of each bicodon, respectively. (XLS 587 kb)

## Abbreviations

ADHD: Attention-deficit/hyperactivity disorder
GWAS: Genome-wide association studies
PA: Protein abundance
RSCU: Relative synonymous codon usage
SNP: Single nucleotide polymorphisms
sSNP: Synonymous single nucleotide polymorphisms
TMD: Temporomandibular joint disorder
