# Supplementary figures and images for: Bicodon bias can determine the role of synonymous SNPs in human diseases
Source: BMC Genomics. 2017 Mar 13;18:227. doi: 10.1186/s12864-017-3609-6 (PMC5347174; doi:10.1186/s12864-017-3609-6)

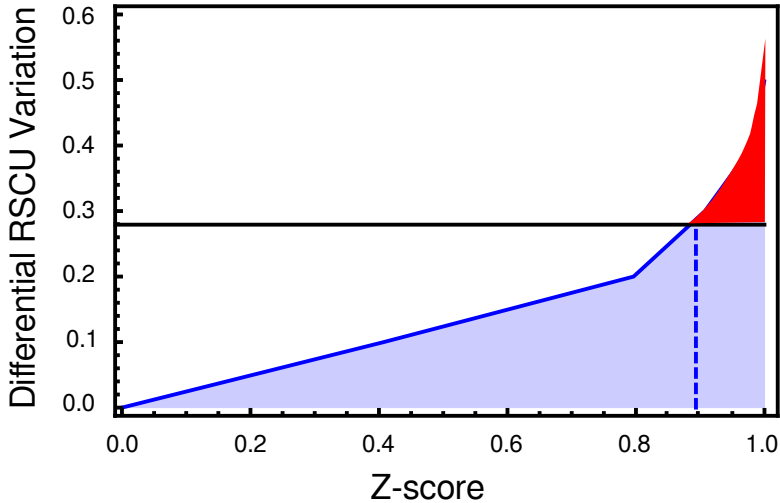

Supplement: Additional file 1 — Figure S1. Z-score function associated with the differential RSCU change of all the synonymous codon variants. The red region indicates the highest 10% pause propensity variation, i.e., those sSNPs with a variation larger than 0.28. (PDF 13 kb) [file 12864_2017_3609_MOESM1_ESM.pdf]

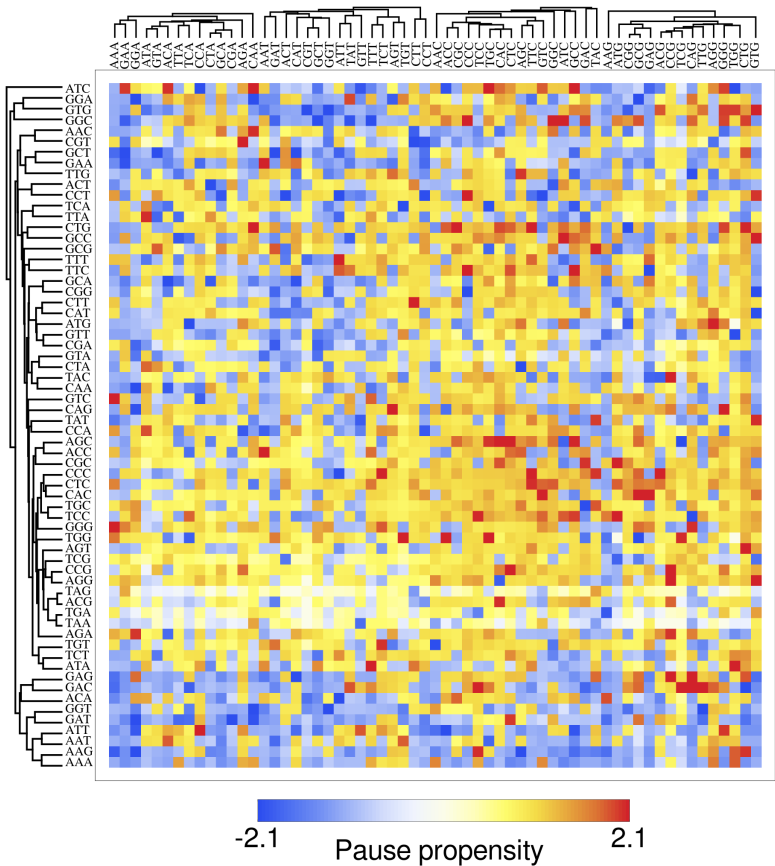

Supplement: Additional file 5 — Figure S2. Pause propensity heat map. The color of each cell is determined by the pause propensity π of the associated bicodons. Columns represent codons corresponding to P-site, while rows represent codons corresponding to A-site, so that each cell in the heat map represents a bicodon. Red cells indicate bicodons with the highest pause propensity value (low PA preference), while blue cells indicate bicodons with the lowest pause propensity value (high PA preference). Rows and columns have been clustered to improve visualization. (PDF 260 kb) [file 12864_2017_3609_MOESM5_ESM.pdf]
