# Supplementary material for: Bicodon bias can determine the role of synonymous SNPs in human diseases
Source: BMC Genomics. 2017 Mar 13;18:227. doi: 10.1186/s12864-017-3609-6 (PMC5347174; doi:10.1186/s12864-017-3609-6)
Supplement: Additional file 2 — Text. Additional details for each disease or clinical trait listed in Table 1, such as basic background, references, accession number and local sequence context analysis. (PDF 88 kb) [file 12864_2017_3609_MOESM2_ESM.pdf]

Additional file 2 for

Bicodon bias can determine the role of synonymous SNPs in human diseases.

by C.B. McCarthy, A. Carrea and L. Diambra
E-mail: ldiambra@gmail.com

Synonymous, but not silent, mutations in human diseases

While changes in protein conformation have been suggested as the underlying mechanism responsible for synonymous mutation-associated disorders, studies that have been able to confirm these changes, or protein function differences, are scarce and the case of *MDR1* gene is the most conclusive one. However, the analysis presented here adds suggestive evidence supporting the fact that sSNPs that do not alter splicing processes neither mRNA structure, but are associated with diseases, could be involved in the alteration of the ribosome-mediated translational attenuation program encoded by bicodons. In this paper, we analyzed the local sequence context of each compiled sSNP, by computing the pause propensity change due to the sSNP in both bicodons affected by the variation. The results of this analysis are summarized in Table 1, and further details for each disease are given below and in the Additional file 3 (Table S1).

Age-related macular degeneration

The main cause of irreversible visual loss in the older (over 60 years) Western population is age-related macular degeneration (AMD) [1]. There is considerable evidence which proves that AMD has a strong genetic component, including the discovery of polymorphisms in genes involved in the regulation of the immune-mediated complement pathway, such as complement factor. In a previous study, in which CFH related 5 (*CFHR5*) gene was analyzed, it was found that the sSNP rs34533956 (C>T at 635 in NM_030787.3) was less frequent in AMD patients, compared with normal controls [2]. By means of bioinformatic tools, the variant was not predicted to create or destroy splice donor or acceptor sites. The authors suggested that this variant is associated with a reduced risk for developing AMD [2].

We performed a local-sequence context analysis of the variant rs34533956. The analysis indicated that the pause propensity value for this variant varies from $\pi = 0.03$, in

GACGTG, to π= -0.76, in GATGTG (Z-score=0.91). However, the Z-score associated with the change in the differential RSCU was only 0.51. Hence, our results suggest that the protective effect of the variant could be associated with the introduction of a fast bicodon (GACGTG) in place of a slow one (GATGTG), thus influencing co-translational folding with the consequent reduced risk for developing AMD.

Longevity

Because telomere shortening is associated with aging in organisms [3, 4], telomere maintenance genes could also be longevity genes, particularly those that regulate telomerase activity. The two major telomerase genes are: *TERC*, which encodes the subunit of the enzyme that provides the template for the synthesis of the TTAGGG repeats, and *TERT*, which encodes its catalytic subunit [5]. In particular, Atzmon *et al.* reported that Ashkenazi centenarians have a longer leukocyte telomere length and a higher frequency of rare *TERT* variants, in comparison with younger controls that did not have a family history of extreme longevity [6]. Moreover, significant associations were found for TERT-rs33954691 with longevity: the T allele and both C|T and T|T genotypes were significantly enriched in centenarians [6]. Even though Sauna *et al.* indicated that TERT-rs33959226 was associated with exceptional longevity and telomere length [7], we did not find any studies reporting this association.

Our local-sequence context analysis of rs33954691 established a pause propensity value of π= -0.01, for the CACGCA bicodon, and a low pause propensity value (π= -0.60) for the CATGCA bicodon. This implies that the bicodon variant CA**C**GCA → CA**T**GCA is involved in a large relative change of pause propensity (Z-score=0.84), whereas the Z-score associated with the differential RSCU variation was 0.71. Our results suggest that there is a considerable alteration of the translational attenuation program encoded by bicodons, which could be related to the higher longevity associated with C|T and T|T. Similarly, our local-sequence context analysis of rs33959226 established a low pause propensity value of π= -0.48, for the GCAGAG bicodon, and a pause propensity value of π= 0.09, for the GCGGAG bicodon. This means that the bicodon variant GC**A**GAG → GC**G**GAG is involved in a large pause propensity change (Z-score=0.83), whereas the change in the differential RSCU was small (Z-score=0.55). This result also suggests that the sSNP rs33959226 might be associated with longevity through an alteration of the translational attenuation program encoded by bicodons.

Asthma

A recent study by Kim and colleagues (2010) [8] identified a new association between asthma and the *SLC6A7* gene, which encodes a neurotransmitter transporter. This study found that the sSNP rs2240794 (T>C at 1565 in NM_014228.3) was infrequently associated with asthma. Furthermore, when atopic status in asthmatics was taken into account, the minor allele of rs2240794 decreased bronchodilator response. In addition, the authors

suggested that the observed variable frequency of the amino acid Asp in the transmembrane alpha helix region, which depends on which of the two synonymous codon is present, was related to differences in tRNA expression levels. These differences, in turn, could affect SLC6A7 activity and, consequently, the susceptibility to asthma [8].

Our analysis of rs2240794 indicated that the relative changes in pause propensity were small (Z-score=0.11 and 0.09), whereas the Z-score associated with the change in the differential RSCU was 0.51. On the other hand, the frequency usage over all coding sequences of the GACAGC bicodon is around 6-fold greater than the frequency usage of the GATAGC bicodon, independently of protein abundance. This difference in the frequency usage of bicodons cannot be explained by the frequency usage of codons GAC and GAT, which are quite similar. Our result suggests that the difference in activity of the protein could be associated with the introduction of a more frequent bicodon in place of a rare one, even though we are not able to relate the rareness of the GATAGC bicodon to a modification of the translational attenuation program. In any case, the alteration introduced by the sSNP rs2240794 is more evident in the frequency usage of bicodons, rather than that of single codons.

Pulmonary sarcoidosis

In a study performed by Sato and colleagues [9], it was found that two polymorphisms in the *NOD2* gene, also known as *CARD15*, present an association with severe pulmonary sarcoidosis phenotypes. In particular, the CARD15 rs1861759 polymorphism (T>G at 1866 in NM_022162.2) was related to better lung function, which suggested that it could be linked to a favorable phenotype. The authors highlighted that, even though it was previously hypothesized that synonymous polymorphisms could affect the timing of co-translational folding with consequent alterations in the structure of protein binding sites [10], further functional studies were needed to confirm this for CARD15 rs1861759.

In our local-sequence context analysis we considered two pairs of bicodons associated with rs1861759: CG**T**GCC → CG**G**GCC, and GTGCG**T** → GTGCG**G**. In both cases the pause propensity values vary considerably: from π= 0.03 in CGTGCC, to π= -0.45 in CGGGCC (Z-score=0.76); and from π= -0.15 in GTGCGT, to π= -1.73 in GTGCGG (Z-score=0.99), whereas the change in the differential RSCU was small (Z-score=0.17). Moreover, in both cases the resulting bicodons correspond to fast bicodons. Our results thus suggest that the favorable phenotype of this variant could be associated with the introduction of faster bicodons, thereby influencing the co-translational folding process.

Tuberculosis meningitis

A study carried out on a Vietnamese adult cohort demonstrated that the sSNP rs7932766 (C>T at 994 in NM_001039661.1) in the Toll-interleukin 1 receptor domain containing adaptor protein (*TIRAP*) gene, is associated with tuberculosis meningitis (TBM) [11]. The authors suggested that this sSNP affects TBM susceptibility through a modulation of

the inflammatory response, since they found that the T|T genotype was associated with decreased whole-blood interleukin-6 production, compared with the C|C genotype.

As in the previous case, in our local-sequence context analysis we considered two pairs of bicodons associated with rs7932766: GC**C**TAC → GC**T**TAC, and GCTGC**C** → GCTGC**T**. In both cases the pause propensity values vary considerably: from π= -0.39 in GCCTAC, to π= 0.06 in GCTTAC (Z-score=0.74); and from π= 0.12 in GCTGCC, to π= -0.28 in GCTGC**T** (Z-score=0.69). Therefore, this sSNP promotes two moderately large, and opposite, changes in the translational attenuation program. However, with the available information we are not able to discern the individual contribution of each one to a higher susceptibility to TBM.

Cystic fibrosis

Various studies have focused on different nucleotide changes in the cystic fibrosis transmembrane conductance regulator (*CFTR*) gene, associated with cystic fibrosis (CF). In one of these studies it was found that synonymous mutations induce exon 12 skipping, leading to reduced levels of normal transcripts [12]. Another study showed that the synonymous variant 2811 G>T causes aberrant splicing [13]. Nevertheless, the most frequent cause of CF is the deletion of three nucleotides (CTT) in the *CFTR* gene. This deletion includes the last cytosine (C) of isoleucine 507 codon (Ile507-ATC), and the two thymidines (T) of phenylalanine 508 codon (Phe508-TTT). The consequences of this deletion are the loss of phenylalanine at position 508 of the CFTR protein (ΔF508), a synonymous codon change in isoleucine 507 (Ile507-ATT), and protein misfolding. It has been demonstrated that the change Ile507-ATC → Ile507-ATT alters the ΔF508 CFTR mRNA structure and translation dynamics, contributing to co-translational misfolding, endoplasmic reticulum-associated protein degradation, and functional defects associated with ΔF508 CFTR [14, 15, 16]. Since the translational rate and protein expression levels of the variant (Ile507-ATC) ΔF508 CFTR improved compared with the native (Ile507-ATT) ΔF508 CFTR, the authors concluded that the reduced translational rates were due to differences in the mRNA secondary structures between these two ΔF508 CFTR mutants (native and variant), rather than the rarity of the ATT codon [14]. In a subsequent study, the same authors confirmed structural differences between the synonymous Ile507-ATT and Ile507-ATC ΔF508 CFTR variants, and found that these protein-folding differences were accompanied by altered CFTR ion channel properties [15]. Summarizing, the alteration in the mRNA secondary structure, derived from codon choice in Ile507, is important in determining the final protein conformation and function.

Our local-sequence context analysis of the variant ΔF508 CFTR, resulted in a pause propensity change from π= -0.05, associated with AT**C**ATC bicodon, to π= -0.01, associated with AT**A**ATC bicodon (Z-score=0.09). A similar analysis for the second case (AATAT**C** → AATAT**A**) established also a small pause propensity change (Z-score=0.22). The change in the differential RSCU was also small (Z-score=0.17). Thus, our results are

consistent with the hypothesis that reduced translational rates are the result of the mRNA secondary structure differences, rather than an alteration of the translational attenuation program encoded by bicodons.

Coeliac disease

One variant within the *CD44* gene (sSNP rs1071695) has been linked to an inherited haplotype in a family with coeliac disease (CD) [17]. Using bioinformatic tools, the authors found that this variant might have an effect on RNA secondary structure, but did not identify any exonic splicing silencers (ESS) or exonic splicing enhancers (ESE), both in normal and in mutated alleles. Furthermore, *in vitro* analysis failed to show any effect of this variant on the CD44 pre-mRNA splicing [17].

Our local-sequence context analysis of the variant rs1071695 established that the CACGTG bicodon has π= 0.27, i.e., it is associated with a high pause propensity value; whereas the CACGTG bicodon, with π= -0.33, is associated with a low pause propensity value. This means that these bicodons are related to a large relative change in the pause propensity (Z-score=0.84). On the other hand, the Z-score associated with the differential RSCU was 0.71. Thus, these results suggest that the sSNP rs1071695 could increase the risk of CD due to a large change in the pause propensity. This, in turn, can alter the ribosome-mediated translational attenuation program, affecting the co-translational folding process, as in the P-glycoprotein case (see below). Another variant that has been associated with this same disease, but within the *APIP* gene, is rs1571133 [17]. Nevertheless, the authors did not find that this variant was linked with the inherited haplotype.

Our analysis of the bicodons associated with variant rs1071695 indicated that their π values are quite similar (ACACTT: π= -0.02; ACCCTT π=0.13), rendering a small relative change in pause propensity (Z-score=0.32). Hence, our results indicate that the rs1571133 variant is not linked with a local alteration of the translational attenuation program of the *APIP* gene.

Crohn's disease

Sequence variants of the autophagy-associated immunity-related GTPase family M protein (IRGM) have been associated with Crohn's disease [18]. Moreover, it has been shown that the synonymous variant rs10065172 in *IRGM* C>T at 1426 in NM_001145805.1, causes an alteration in a binding site for miR-196. This, in turn, causes a deregulation of IRGM-dependent xenophagy in Crohn's disease [19]. In particular, this synonymous variant has been strongly associated with Crohn's disease in individuals of European descent [18, 20, 21], and in an Asian population [22].

We analyzed the pause propensity associated with the bicodons CTGATG and ATGATG, involved in the aforementioned sSNP. In this case, the Z-scores associated with pause propensity changes were 0.77 and 0.02, whereas the change in the differential RSCU was high (Z-score=0.93). The small change in the pause propensity associated with this

sSNP is in agreement with the above mentioned fact that this synonymous mutation operates by altering a binding site for miR-196, and not by altering the translational attenuation program mediated by bicodons.

Smoking-related cancer

Nibrin is the product of the *NBS1* gene, and it plays a critical role in the cellular response to DNA damage by means of forming the hMRE11-hRAD50-NBS1 (MRN) nuclease complex. Its expression was found to be increased in lung and upper aerodigestive tract (UADT) tumor tissues [23]. Several studies of lung [24, 25, 23] and UADT cancers [25, 26] have related *NBS1* SNPs to cancer susceptibility. This could occur through the functional change of the MRN nuclease complex, which results in a diminished DNA repair ability [23]. *NBS1* rs709816 (T>C at 1307 in NM_002485.4) was positively associated with bladder cancer [25], and the carriage of its variant C allele by large cell lung carcinoma patients was related to shorter survival [27]. Considering the smoking status, rs709816 was positively associated with smoking-related cancers [25]. On the other hand, the association of *NBS1* rs1061302 (A>G at 2126 in NM_002485.4) with lung cancer varies depending on the smoking status. For never-smokers the authors found a dose-response relationship for the variant allele and lung cancer, while for smokers an inverse relationship between rs1061302 heterozygotes and lung cancer was found [25]. In another study, consistent associations between the G|G genotype and lower survival of smokers with non-small cell lung cancer (NSCLC), squamous cell lung carcinoma, or small cell lung carcinoma, were found [27]. It was suggested that MRN binding ability and DNA double-strand break (DSB) repair function could be impaired due to the location of *NBS1* rs1061302 at the binding domain of MRN complex. This could be the link between this sSNP and carcinogenesis and cancer progression [25].

Our analysis of rs709816 indicated that the pause propensity value changes from $\pi=$ -0.42, in GATGCA, to $\pi=$ 0.11, in GACGCA. This means a large relative change in the pause propensity (Z-score=0.8), whereas the change in the differential RSCU was only 0.13 (Z-score=0.51). Thus, these results suggest that the higher risk for developing smoking-related cancers associated with the variant C allele, could be associated with the introduction of a slow bicodon in place of a fast one, rather than with a rare codon. This, in turn, would affect the co-translational folding process.

With respect to rs1061302, our local-sequence context analysis indicated that the pause propensity values associated with this variant vary significantly (from $\pi=$ -0.46, in AATCCA, to $\pi=$ 0.06, in AATCCG; and a resultant Z-score=0.79), whereas the change in the differential RSCU was small (Z-score=0.47). On the other hand, frequency usage of AATCCA bicodon was around 6-fold greater than the AATCCG bicodon. It has been proposed that rs1061302 could affect proper binding of the MRN complex and also modify its ability to accurately repair or detect DNA DSBs. One reason for this is that the altered nucleotide could affect the translational rate [25]. Our results strongly suggest that this

alteration could be associated with the introduction of a rare bicodon in place of a more frequent one, which is also a faster bicodon.

Colorectal cancer

The nucleotide excision repair (NER) complex is a group of proteins that are able to repair DNA damage, and the excision repair cross-complementing 1 (*ERCC1*) gene codes for a NER protein. Because of this, polymorphisms in *ERCC1* may alter the DNA repair capacity, leading to different biological responses to DNA damage [28]. In particular, the sSNP rs11615 (C>T at 542 in NM_202001.2) of *ERCC1* has been related to increased colorectal cancer risk [28], and it was also associated with the clinical outcome of colorectal cancer (CRC) patients treated with oxaliplatin-based chemotherapy [29]. Notably, this latter study showed that the T allele was associated with a reduced response to chemotherapy in Asians, and with a significant increase in the risk for shorter progression free survival (PFS) and overall survival in all patients [29]. Moreover, Hou and colleagues found that patients carrying the rs11615 T|T genotype and T allele had a marginally increased risk of CRC, when compared with those with the C|C genotype [28].

Our local-sequence context analysis of rs11615 indicated that the relative change in the pause propensity was high (Z-score=0.97), i.e., the π value of bicodons vary significantly: from π= -1.12, in AATGTG, to π= -0.04, in AACGTG. On the other hand, the change in the differential RSCU was only 0.09 (Z-score=0.07). Hence, our results suggest that the increased risk for CRC and reduced chemotherapy response associated with the T allele and T|T genotype, could be caused by a different protein conformation induced by the introduction of a fast bicodon in place of a slow one.

Chronic myeloid leukemia

In patients with chronic myeloid leukemia (CML) a translocation juxtaposes the *BCR* gene on to the *ABL1* gene (BCR-ABL), and the resulting fusion protein acts as an oncogene with deregulated tyrosine kinase activity [30]. In a recent study, CML patients were screened for BCR-ABL kinase domain mutations, and a SNP analysis was performed on normal, non-translocated ABL alleles [31]. ABL rs2229069 and rs2227985 were detected in the CML patients, but the clinical impact of these sSNPs is unknown [31].

Our local-sequence context analysis of these sSNPs established that the bicodon variant CGCAC**G** $\rightarrow$ CGCAC**A**, associated with the first sSNP, is involved in a large pause propensity change (Z-score=0.80), whereas the change in the differential RSCU is low (Z-score=0.24). This suggests that rs2229069 could promote a significant alteration of the translational attenuation program encoded by bicodons, with possible clinical outcomes. On the other hand, a similar analysis of rs2227985 established a low pause propensity (π= -0.13) for the CAGGAA bicodon, and a pause propensity value of π=0.28, for the CAGGAA bicodon. This implies that this bicodon variant is involved in a moderate pause propensity change (Z-score=0.69), whereas the change in the differential RSCU

was small (Z-score=0.10). This, in turn, suggests that there is an alteration of the translational attenuation program encoded by bicodons, with possible clinical consequences for CML patients.

Non-small-cell lung carcinoma

Gefitinib (IressaTM, ZD1839) is used for the treatment of patients with advanced NSCLC which are refractory to chemotherapy. It selectively targets the epidermal growth factor receptor (EGFR) and blocks its tyrosine kinase activity [32]. Nevertheless, the response rates to Gefitinib among advanced NSCLC patients vary greatly [33, 34, 35], and a significant portion of this interindividual variability is due to genetic variations [36, 37]. The sSNP rs2293347 (C>T at 3228 in NM_005228.3) in *EGFR* has been associated with the efficacy of Gefitinib. In particular, the favorable rs2293347 C|C genotype has been related with a higher response rate and longer PFS, in comparison with the unfavorable EGFR rs2293347 T|T or C|T genotypes [38, 39].

Our local-sequence context analysis of rs2293347 showed that bicodons affected by this sSNP have a preference for codifying highly abundant proteins. In line with this, the pause propensity change from π= -0.32, in ACAGAC, to π= -0.09, in ACAGAT, indicates a small relative change in the pause propensity (Z-score=0.48). The change in the differential RSCU was also small (Z-score=0.51). Our results thus suggest that the increased risk associated with the T|T and T|C genotypes would not be caused by an alteration of the translational attenuation program encoded by bicodons.

Cervical and vulvar cancer

Interleukin 2 (IL2) is a key component of the adaptive immune response to the human papillomavirus (HPV) infection, with the subsequent development and growth of tumors driven by the viral oncogenes [40, 41]. Furthermore, the sSNP rs2069763 (G>T at 169 in NM_000586.3) in the *IL2* gene has been associated with cervical and vulvar cancer risk. In particular, the rs2069763 T|T genotype was associated with a reduced risk of cervical and vulvar cancer [42].

Our local-sequence context analysis of rs2069763 showed that bicodons affected by this sSNP have a preference for codifying lowly abundant proteins. In line with this, the pause propensity change from π= 0.69, in CTGCTG, to π= 0.45, in CTTCTG, indicates a small relative change in the pause propensity (Z-score=0.49). Our results thus suggest that the reduced risk associated with the T|T genotype would not be caused by an alteration of the translational attenuation program encoded by bicodons. Nevertheless, the frequency usage of the CTGCTG bicodon is four-fold higher than the frequency usage of the CTTCTG bicodon.

Multidrug resistance

It was recently shown that the sSNP rs1045642 in the multidrug resistance 1 (*MDR1*) gene (T>C at 3928 in NM_000927.4) resulted in P-glycoprotein (P-gp) with altered drug and inhibitor interactions [10]. The authors proposed that the introduction of a rare codon (Ile-ATC) influenced co-translational folding, thereby altering the structure of both the substrate and inhibitor interaction sites [10]. Furthermore, a subsequent study by the same group confirmed altered protein conformation, stability, and transporter specificity in epithelial monolayers [43]. Summarizing, rs1045642 is a sSNP that alters protein structure and function, contributing to a human disorder.

The sSNP rs1045642 is associated with a low change in the differential RSCU (Z-score=0.55). On the other hand, our local-sequence context analysis established a low pause propensity value (π= -0.81) for the ATTGTG bicodon, and a high pause propensity value (π= 0.11) for the ATCGTG bicodon. This implies that the bicodon variant AT**T**GTG $\rightarrow$ AT**C**GTG is involved in a large pause propensity change (Z-score=0.94). This change is not reflected by the frequency usage of both bicodons over all coding sequences, independently of protein abundance, which is approximately the same. Furthermore, Kimchi-Sarfaty *et al.* observed that the haplotype corresponding to ATAGTG bicodon, also synonymous, is associated with a greater decrease in the inhibitory effect than the ATTGTG haplotype [10]. This is in agreement with our observations, since the ATAGTG bicodon also has an associated low pause propensity value, $\pi = -0.56$. These results suggest that the altered protein folding underlies the large pause propensity change of bicodons, rather than the rarity of the ATC codon, as was previously proposed by Kimchi-Sarfaty *et al.*.

Alzheimer's disease

The cholinergic receptor alpha 4 (*CHRNA4*) gene encodes the neuronal nicotinic acetylcholine receptor alpha-4 subunit, which has been associated with Alzheimer's disease (AD) [44]. Various studies have shown that the variation rs1044396 (C>T at 1860 in NM_000744.6) in this gene affects attention and negative emotionality in normal adults [45, 46, 47]. Furthermore, a recent study linked this polymorphism with depression and loneliness in the aged. In particular, subjects with the rs1044396 C|C genotype had higher levels of depression compared with the T-allele carriers [48].

Our local-sequence context analysis of CHRNA4-rs1044396 resulted in similar values of pause propensity change in all bicodons affected by this sSNP, which means small changes in the pause propensity values (Z-score=0.26 and Z-score=0.07). The change in the differential RSCU was 0.2 (Z-score=0.74). This result suggests that AD would not be linked to an alteration of the translational attenuation program encoded by bicodons in the case of rs1044396.

Cytochrome oxidase (COX) activity varies between individuals and most people with AD have systemically low or below normal range COX activities [49]. Other three sSNPs

associated with genes involved in COX activity were analyzed in detail for AD: COX6B1-rs7991, COX6C-rs1130569, and COX8A-rs61759492.

Our local-sequence context analysis of COX6B1-rs7991 established a pause propensity of π=-0.57 for the AAGACC bicodon, and a pause propensity of π=0.02, for the AAGACT bicodon. This implies that the bicodon variant AAGAC**C** $\rightarrow$ AAGAC**T** is involved in a large pause propensity change (Z-score=0.84), whereas the change in the differential RSCU was small (Z-score=0.36). This result suggests that there is a significant alteration of the translational attenuation program of COX6B1 due to rs7991, which could be related to AD.

A similar analysis of COX8A-rs61759492 established that the bicodon variant AT**C**ATG $\rightarrow$ AT**A**ATG is also involved in a large pause propensity change of (Z-score=0.83) Furthermore, the ATCATG bicodon is six-fold more frequently used than the ATAATG bicodon. As with COX6B1-rs7991, this result suggests that there is a noteworthy alteration of the translational attenuation program of COX8A due to rs61759492, which could contribute to AD development.

On the other hand, the local-sequence context analysis of COX6C-rs1130569 resulted in similar values of pause propensity change in all bicodons affected by this sSNP, which means a small change in the pause propensity value (Z-score=0.56). Hence, our result suggests that there is not a significant alteration of the translational attenuation program of COX6C due to rs1130569.

Attention-deficit/hyperactivity disorder

Neurotrophic factors, which participate in neuronal survival and synaptic efficiency, have been involved in attention-deficit/hyperactivity disorder (ADHD), a neurodevelopmental disorder [50, 51, 52, 53, 54]. In particular, neurotrophin-3 (*NTF3*) rs6332 (G>A at 502 in NM_001102654.1) has been associated with childhood ADHD [53], the A-allele has been suggested as a risk factor for ADHD susceptibility [52], and the A allele has also been associated with increased emotional side effects in response to methylphenidate treatment in children with ADHD [54].

Our local-sequence context analysis of rs6332 resulted in similar values of pause propensity change in all bicodons affected by this sSNP, which means small changes in the pause propensity (Z-score=0.20 and Z-score=0.46). The change in the differential RSCU was also small (Z-score=0.47). Our results thus suggest that the higher risk factor for ADHD associated with the A allele would not be due to an alteration of the translational attenuation program encoded by bicodons.

Huntington's disease

The adenosinergic A2A receptor (ADORA2A) is a G-protein-coupled receptor that is expressed preferentially within the striatum of the brain [55], and several studies relate A2A receptors with Huntington's disease (HD) pathogenesis [56, 57]. In particular,

ADORA2A-rs5751876 was found to influence age at onset in HD, with T|T genotype patients showing an earlier age at onset in HD compared to patients with C|C or C|T genotypes [58, 59].

We analyzed two sets of bicodons for rs5751876 (T>C at 1542 in NM_000675.5). In the first case (TA**T**GCC → TA**C**GCC), our local-sequence context analysis established a low pause propensity value (π=-0.98) for the TATGCC bicodon, and a high pause propensity value (π=0.21) for the TACGCC bicodon. This implies that variant TA**T**GCC → TA**C**GCC is involved in a large pause propensity change (Z-score=0.98). Furthemore, in the second case (GGCTA**T** → GGCTA**C**), a similar analysis established a high pause propensity value (π=0.04) for the GGCTAT bicodon, and a low pause propensity value (π=-1.26) for the GGCTAC bicodon, which implies that this bicodon variant is also involved in a larger pause propensity change (Z-score=0.99). On the other hand, the change in the differential RSCU is small (Z-score=0.24).

These results suggest that, for both sets of bicodons, there is a significant alteration of the translational attenuation program encoded by bicodons, which in turn could be associated with the later age at onset in HD in patients with the C|C or C|T genotypes.

Schizophrenia

It is thought that altered dopamine function underlies several of the symptoms of schizophrenia, as well as the action of antipsychotic medication [60, 61]. The dopamine signaling pathway gene, dopamine receptor D2 (*DRD2*), codes for the main target of dopamine and of antipsychotic drugs [62]. Polymorphisms in this gene have repeatedly been associated with schizophrenia [63, 64, 65]. In particular, the sSNP rs6277 in *DRD2* has been positively associated with schizophrenia, with higher frequencies of the C allele and the C|C genotype in patients with schizophrenia [66, 67, 68, 69, 70]. Furthermore, it has been shown that the T allele causes decreased mRNA translation and stability, and a weakened response to dopamine-induced up-regulation of *DRD2* [71]. On the other hand, DRD2-rs6275 was also found to predict disease risk among schizophrenia patients, together with catechol-O-methyl transferase (COMT)-rs4680 [62].

We analyzed two sets of bicodons for rs6277. In the first case (CC**C**GAC → CC**T**GAC), our local-sequence context analysis established a high pause propensity value (π= 0.27) for the CCCGAC bicodon, and a low pause propensity value (π= -0.18) for the CCTGAC bicodon. Likewise, in the second case (ACTCC**C** → ACTCC**T**), our local-sequence context analysis established a high pause propensity value (π= 0.41) for the ACTCCC bicodon, and a low pause propensity value (π= -0.53) for the ACTCCT bicodon. This implies that this bicodon variant is involved in an even larger pause propensity change (Z-score=0.95), while the change in the differential RSCU was also high (Z-score=0.82). These results suggest that the transitions from a slow bicodon to a fast bicodon, in both bicodon variants, due to rs6277, could constitute a significant alteration of the translational attenuation program encoded by bicodons. This, in turn, could be associated with

the weakened response to dopamine-induced up-regulation of *DRD2* due to the T allele.

A similar analysis of sSNP rs6275 established a pause propensity value of π= 0.24, for the CATGGT bicodon, and a pause propensity value of π= -0.10, for the CACGGT bicodon. Likewise, in the second case (CACCA**T** → CACCA**C**), our local-sequence context analysis established a pause propensity value of π= 0.17, for the CACCAT bicodon, and a high pause propensity value (π= 0.71) for the CACCAC bicodon. This implies that this bicodon variant is involved in a larger pause propensity change (Z-score=0.81), whereas the change in the differential RSCU was moderately high (Z-score=0.71). This result suggests that the alteration of the translational attenuation program due to rs6275, could be related to the higher risk of schizophrenia that has been associated with this sSNP.

Peptidylarginine deiminase 2 (*PADI2*) is expressed throughout the nervous system and has been implicated in neurodegeneration [72, 73], suggesting that it is probably related to the pathogenesis of neuropsychiatric diseases that involve neurodegenerative processes. However, a recent study found that PADI2-rs2076615 is not related to genetic susceptibility to schizophrenia [74].

We analyzed two sets of bicodons for rs2076615. In the first case (GG**T**GGC → GG**G**GGC), our local-sequence context analysis established a low pause propensity value (π= -0.69) for the GGTGGC bicodon, and a pause propensity value of π= 0.14, for the GGGGGC bicodon. This implies that the bicodon variant is involved in a large pause propensity change (Z-score=0.93), whereas the change in the differential RSCU was moderate (Z-score=0.77). These results suggest that the sSNP rs2076615 could cause a significant alteration of the translational attenuation program encoded by bicodons.

Synaptogyrin 1 gene (*SYNGR1*) plays a key role in neurotransmitter release and synaptic plasticity [75, 76]. Reduced expression of *SYNGR1* has been reported in the prefrontal cortex of patients with schizophrenia [77]. Furthermore, two studies associated a nonsense mutation (Try27Ter) and five SNPs in *SYNGR1* with the disease [78, 79]. Nevertheless, even though SYNGR1-rs74681509 was associated with schizophrenia in [7], we did not find any studies reporting this association.

We analyzed two sets of bicodons for rs74681509. In the case of ACCTT**C** → ACCTT**T**, our local-sequence context analysis established a pause propensity value of π=0.31, for the TTCGAC bicodon, and a pause propensity value of π=-0.28, for the TTTGAC bicodon. This implies that this bicodon variant is involved in a large pause propensity change (Z-score=0.84), whereas the change in the differential RSCU was small (Z-score=0.36). These results suggest that, for both sets of bicodons, there is a significant alteration of the translational attenuation program encoded by bicodons.

Painful temporomandibular joint disorder

The COMT enzyme not only metabolizes catecholamines (dopamine, epinephrine and norepinephrine), but also plays a key role in the modulation of dopaminergic and adren-

ergic neurotransmission [80]. Different studies found that genetic variations in the *COMT* gene substantially influence pain sensitivity and are implicated in the risk of developing temporomandibular joint disorder (TMD) [81]. Three common haplotypes of this gene, divergent in two synonymous (rs4633, rs4818) and one non-synonymous SNPs (rs4680), code for differences in COMT enzymatic activity and were associated with pain sensitivity. In particular, the haplotypes that diverged in the synonymous changes were the ones that exhibited the largest difference in COMT enzymatic activity, due to a reduced amount of translated protein. Furthermore, the lowest protein levels and enzymatic activity were associated with the most stable mRNA secondary structure (Nackley et al., 2006). Furthermore, the sSNP rs769223 (G>A at 651 in NM_000754.3) has been associated to TMD [7, 82], even though this SNP has not yet been validated by 1000Genomes.

We analyzed two sets of bicodons affected by the variant rs769223. In the first case (GC**G**AGG → GC**A**AGG), our local-sequence context analysis established a pause propensity value of π= -0.07, for the GCGAGG bicodon, and a pause propensity value of π= 0.32 for the GCAAGG bicodon. This implies that this bicodon variant is involved in a moderate change of the pause propensity (Z-score=0.67). A similar analysis of the bicodon variant GGGGC**G** → GGGGC**A** resulted in a small pause propensity change (Z-score=0.38), whereas the change in the differential RSCU was moderate (Z-score=0.55). These results suggest that there is not a significant alteration of the translational attenuation program in agreement to the fact that known mRNA secondary structure modification.

Type III hyperlipidemia

Lipoprotein lipase (LPL) is a key enzyme in the metabolism of triglycerides, and various studies have associated variants in its gene with triglyceride levels [83, 84, 85, 86]. A recent study analyzed the frequency of rare variants in the *LPL* gene in patients with various forms of hypertriglyceridemia (HTG) [87]. The authors found that rare variants in the *LPL* gene are frequent in patients with severe and moderate HTG, but do not play a role in the development of Type III hyperlipidemia (HLP). In particular, LPL-rs45607438, rs316, rs1121923, and rs248 were found in the analyzed HTG patients, but were not associated with the HLP disorder [87].

We performed a local-sequence context analysis of the sSNPs mentioned above. It established large pause propensity changes in the following bicodon variants: CA**T**GTT → CA**C**GTT (Z-score=0.89) associated with rs45607438, GT**G**GCC → GT**A**GCC (Z-score=0.89) associated with rs1121923, and AAGAC**C** → AAGAC**A** (Z-score=0.91) associated with rs316. The differential RSCU changes in these sSNPs were all small.

In the case of rs248, our analysis did not show a significant change in the pause propensity. These results suggest that the different activities of the LPL enzyme could be associated with a significant alteration of the translational attenuation program due to either rs45607438 or rs316.

Chronic hepatitis C

Interferon regulatory factor 7 (*IRF7*) rs1061501 (G>A at 776 in NM_004031.2) has been associated with abnormally low amounts of platelets in chronic hepatitis C patients receiving a combination therapy of interferon plus ribavirin [88]. Nevertheless, the mechanism by which this sSNP affects the biological function of IFN signalling and contributes to hematological adverse effects in i nterferon-treated patients, remains unknown.

Our local-sequence context analysis of rs1061501 established a low pause propensity value (π= -0.10) for the CGGGAT bicodon, and an even lower pause propensity value (π= -0.68) for the CGAGAT bicodon. This implies that the bicodon variant CG**G**GAT $\rightarrow$ CG**A**GAT is involved in a large relative change of the pause propensity (Z-score=0.84), whereas the cahnge in the differential RSCU was very small (Z-score=0.17). Thus, the notable alteration of the translational attenuation program due to the sSNP rs1061501 could be associated with the cytopenia observed in interferon-treated patients.

Osteoporosis

It has been suggested that the sSNP rs11033026 in exon 9 of the *CD44* gene could increase susceptibility to osteoporosis through the alteration of the splicing mechanism [89]. In that study a preliminary bioinformatic analysis suggested that the presence of the A allele, located 32 base pairs upstream from the exon-intron junction, eliminates an ESE site, wich could possibly lead to an alteration of the RNA splicing process.

We performed a local-sequence context analysis of the variant rs11033026 and found the CATGAG bicodon is associated with a low pause propensity value (π= -0.49), whereas the CATGAA bicodon is associated with a high pause propensity value (π= 0.37). The change in the differential RSCU was small (Z-score=0.10). These results indicate that the large change observed in the pause propensity (Z-score=0.93) could result in an alteration of the translational attenuation program, with consequences on the co-translational folding process.

References

1. E. M. Stone. Macular degeneration. *Annu Rev Med*, 58:477–90, 2007.

2. U. Narendra, GJT. Pauer, and S.A. Hagstrom. Genetic analysis of complement factor h related 5, cfhr5, in patients with age-related macular degeneration. *Mol. Vis.*, 15:731–736, 2009.

3. J. Lindsey, N. I. McGill, L. A. Lindsey, D. K. Green, and H. J. Cooke. In vivo loss of telomeric repeats with age in humans. *Mutat Res*, 256(1):45–8, 1991.

4. P. E. Slagboom, S. Droog, and D. I. Boomsma. Genetic determination of telomere size in humans: a twin study of three age groups. *Am J Hum Genet*, 55(5):876–82, 1994.

5. E. H. Blackburn, C. W. Greider, and J. W. Szostak. Telomeres and telomerase: the path from maize, tetrahymena and yeast to human cancer and aging. *Nat Med*, 12(10):1133–8, 2006.

6. G. Atzmon, M. Cho, R. M. Cawthon, T. Budagov, M. Katz, X. Yang, G. Siegel, A. Bergman, D. M. Huffman, C. B. Schechter, W. E. Wright, J. W. Shay, N. Barzilai, D. R. Govindaraju, and Y. Suh. Evolution in health and medicine sackler colloquium: Genetic variation in human telomerase is associated with telomere length in ashkenazi centenarians. *Proc Natl Acad Sci U S A*, 107 Suppl 1:1710–7, 2010.

7. Zuben E Sauna and Chava Kimchi-Sarfaty. Understanding the contribution of synonymous mutations to human disease. *Nature Reviews Genetics*, 12(10):683–691, 2011.

8. J. H. Kim, H. S. Cheong, B. L. Park, J. S. Bae, S. Jung, S. H. Yoon, J. S. Park, A. S. Jang, S. W. Park, S. T. Uh, Y. H. Kim, H. K. Hwang, C. S. Park, and H. D. Shin. A new association between polymorphisms of the slc6a7 gene in the chromosome 5q31-32 region and asthma. *J Hum Genet*, 55(6):358–65, 2010.

9. H Sato, H R T Williams, P Spagnolo, a Abdallah, T Ahmad, T R Orchard, S J Copley, S R Desai, a U Wells, R M du Bois, and K I Welsh. CARD15/NOD2 polymorphisms are associated with severe pulmonary sarcoidosis. *The European Respiratory Journal*, 35(2):324–30, 2010.

10. Chava Kimchi-Sarfaty, Jung Mi Oh, In-Wha Kim, Zuben E Sauna, Anna Maria Calcagno, Suresh V Ambudkar, and Michael M Gottesman. A silent polymorphism in the MDR1 gene changes substrate specificity. *Science*, 315(5811):525–528, 2007.

11. T. R. Hawn, S. J. Dunstan, G. E. Thwaites, C. P. Simmons, N. T. Thuong, N. T. Lan, H. T. Quy, T. T. Chau, N. T. Hieu, S. Rodrigues, M. Janer, L. P. Zhao, T. T. Hien, J. J. Farrar, and A. Aderem. A polymorphism in toll-interleukin 1 receptor domain containing adaptor protein is associated with susceptibility to meningeal tuberculosis. *J Infect Dis*, 194(8):1127–34, 2006.

12. F. Pagani, M. Raponi, and F. E. Baralle. Synonymous mutations in CFTR exon 12 affect splicing and are not neutral in evolution. *Proceedings of the National Academy of Sciences*, 102(18):6368–6372, may 2005.

13. V. Faa, A. Coiana, F. Incani, L. Costantino, A. Cao, and M. C. Rosatelli. A synonymous mutation in the cftr gene causes aberrant splicing in an italian patient affected by a mild form of cystic fibrosis. *J Mol Diagn*, 12(3):380–3, 2010.

14. R. A. Bartoszewski, M. Jablonsky, S. Bartoszewska, L. Stevenson, Q. Dai, J. Kappes, J. F. Collawn, and Z. Bebok. A synonymous single nucleotide polymorphism in deltaf508 cftr alters the secondary structure of the mrna and the expression of the mutant protein. *J Biol Chem*, 285(37):28741–8, 2010.

15. A. Lazrak, L. Fu, V. Bali, R. Bartoszewski, A. Rab, V. Havasi, S. Keiles, J. Kappes, R. Kumar, E. Lefkowitz, E. J. Sorscher, S. Matalon, J. F. Collawn, and Z. Bebok. The silent codon change i507-atc-¿att contributes to the severity of the deltaf508 cftr channel dysfunction. *FASEB J*, 27(11):4630–45, 2013.

16. V. Bali, A. Lazrak, P. Guroji, L. Fu, S. Matalon, and Z. Bebok. A synonymous codon change alters the drug sensitivity of deltaf508 cystic fibrosis transmembrane conductance regulator. *FASEB J*, 30(1):201–13, 2016.

17. C. Vidal, J. Borg, A. Xuereb-Anastasi, and C. A. Scerri. Variants within protectin (cd59) and cd44 genes linked to an inherited haplotype in a family with coeliac disease. *Tissue Antigens*, 73(3):225–35, 2009.

18. M. Parkes, J. C. Barrett, N. J. Prescott, M. Tremelling, C. A. Anderson, S. A. Fisher, R. G. Roberts, E. R. Nimmo, F. R. Cummings, D. Soars, H. Drummond, C. W. Lees, S. A. Khawaja, R. Bagnall, D. A. Burke, C. E. Todhunter, T. Ahmad, C. M. Onnie, W. McArdle, D. Strachan, G. Bethel, C. Bryan, C. M. Lewis, P. Deloukas, A. Forbes, J. Sanderson, D. P. Jewell, J. Satsangi, J. C. Mansfield, L. Cardon, and C. G. Mathew. Sequence variants in the autophagy gene irgm and multiple other replicating loci contribute to crohn's disease susceptibility. *Nat Genet*, 39(7):830–2, 2007.

19. Patrick Brest, Pierre Lapaquette, Mouloud Souidi, Kevin Lebrigand, Annabelle Cesaro, Valérie Vouret-Craviari, Bernard Mari, Pascal Barbry, Jean-François Mosnier, Xavier Hébuterne, Annick Harel-Bellan, Baharia Mograbi, Arlette Darfeuille-Michaud, and Paul Hofman. A synonymous variant in IRGM alters a binding site for miR-196 and causes deregulation of IRGM-dependent xenophagy in Crohn's disease. *Nature genetics*, 43(3):242–5, 2011.

20. S. A. McCarroll, A. Huett, P. Kuballa, S. D. Chilewski, A. Landry, P. Goyette, M. C. Zody, J. L. Hall, S. R. Brant, J. H. Cho, R. H. Duerr, M. S. Silverberg, K. D. Taylor, J. D. Rioux, D. Altshuler, M. J. Daly, and R. J. Xavier. Deletion polymorphism upstream of irgm associated with altered irgm expression and crohn's disease. *Nat Genet*, 40(9):1107–12, 2008.

21. P. Brest, E. A. Corcelle, A. Cesaro, A. Chargui, A. Belaid, D. J. Klionsky, V. Vouret-Craviari, X. Hebuterne, P. Hofman, and B. Mograbi. Autophagy and crohn's disease: at the crossroads of infection, inflammation, immunity, and cancer. *Curr Mol Med*, 10(5):486–502, 2010.

22. CM Moon, DJ Shin, SW Kim, NH Son, A Park, B Park, ES Jung, ES Kim, SP Hong, TI Kim, WH Kim, and JH Cheon. Associations between genetic variants in the irgm gene and inflammatory bowel diseases in the korean population. *Inflamm Bowel Dis.*, 19(1):106–114, 2013.

23. L. Yang, Y. Li, M. Cheng, D. Huang, J. Zheng, B. Liu, X. Ling, Q. Li, X. Zhang, W. Ji, Y. Zhou, and J. Lu. A functional polymorphism at microrna-629-binding site in the 3'-untranslated region of nbs1 gene confers an increased risk of lung cancer in southern and eastern chinese population. *Carcinogenesis*, 33(2):338–47, 2012.

24. B. A. Rybicki and M. C. Iannuzzi. Epidemiology of sarcoidosis: recent advances and future prospects. *Semin Respir Crit Care Med*, 28(1):22–35, 2007.

25. S. L. Park, D. Bastani, B. Y. Goldstein, S. C. Chang, W. Cozen, L. Cai, C. Cordon-Cardo, B. Ding, S. Greenland, N. He, S. K. Hussain, Q. Jiang, Y. C. Lee, S. Liu, M. L. Lu, T. M. Mack, J. T. Mao, H. Morgenstern, L. N. Mu, S. S. Oh, A. Pantuck, J. C. Papp, J. Rao, V. E. Reuter, D. P. Tashkin, H. Wang, N. C. You, S. Z. Yu, J. K. Zhao, and Z. F. Zhang. Associations between nbs1 polymorphisms, haplotypes and smoking-related cancers. *Carcinogenesis*, 31(7):1264–71, 2010.

26. J. Zheng, C. Zhang, L. Jiang, Y. You, Y. Liu, J. Lu, and Y. Zhou. Functional nbs1 polymorphism is associated with occurrence and advanced disease status of nasopharyngeal carcinoma. *Mol Carcinog*, 50(9):689–96, 2011.

27. T. Yang, P. Y. Chang, S. L. Park, D. Bastani, S. C. Chang, H. Morgenstern, D. P. Tashkin, J. T. Mao, J. C. Papp, J. Y. Rao, W. Cozen, T. M. Mack, S. Greenland, and Z. F. Zhang. Tobacco smoking, nbs1 polymorphisms, and survival in lung and upper aerodigestive tract cancers with semi-bayes adjustment for hazard ratio variation. *Cancer Causes Control*, 25(1):11–23, 2014.

28. R. Hou, Y. Liu, Y. Feng, L. Sun, Z. Shu, J. Zhao, and S. Yang. Association of single nucleotide polymorphisms of ercc1 and xpf with colorectal cancer risk and interaction with tobacco use. *Gene*, 548(1):1–5, 2014.

29. S. C. Ma, Y. Zhao, T. Zhang, X. L. Ling, and D. Zhao. Association between the ercc1 rs11615 polymorphism and clinical outcomes of oxaliplatin-based chemotherapies in gastrointestinal cancer: a meta-analysis. *Onco Targets Ther*, 8:641–8, 2015.

30. J. V. Melo and D. J. Barnes. Chronic myeloid leukaemia as a model of disease evolution in human cancer. *Nat Rev Cancer*, 7(6):441–53, 2007.

31. T. Ernst, J. Hoffmann, P. Erben, B. Hanfstein, A. Leitner, R. Hehlmann, A. Hochhaus, and M. C. Muller. Abl single nucleotide polymorphisms may masquerade as bcr-abl mutations associated with resistance to tyrosine kinase inhibitors in patients with chronic myeloid leukemia. *Haematologica*, 93(9):1389–93, 2008.

32. M. Fukuoka, S. Yano, G. Giaccone, T. Tamura, K. Nakagawa, J. Y. Douillard, Y. Nishiwaki, J. Vansteenkiste, S. Kudoh, D. Rischin, R. Eek, T. Horai, K. Noda, I. Takata, E. Smit, S. Averbuch, A. Macleod, A. Feyereislova, R. P. Dong, and J. Baselga. Multi-institutional randomized phase ii trial of gefitinib for previously treated patients with advanced non-small-cell lung cancer (the ideal 1 trial) [corrected]. *J Clin Oncol*, 21(12):2237–46, 2003.

33. M. G. Kris, R. B. Natale, R. S. Herbst, Jr. Lynch, T. J., D. Prager, C. P. Belani, J. H. Schiller, K. Kelly, H. Spiridonidis, A. Sandler, K. S. Albain, D. Cella, M. K. Wolf, S. D. Averbuch, J. J. Ochs, and A. C. Kay. Efficacy of gefitinib, an inhibitor of the epidermal growth factor receptor tyrosine kinase, in symptomatic patients with non-small cell lung cancer: a randomized trial. *JAMA*, 290(16):2149–58, 2003.

34. D. H. Lee, J. Y. Han, H. G. Lee, J. J. Lee, E. K. Lee, H. Y. Kim, H. K. Kim, E. K. Hong, and J. S. Lee. Gefitinib as a first-line therapy of advanced or metastatic adenocarcinoma of the lung in never-smokers. *Clin Cancer Res*, 11(8):3032–7, 2005.

35. R. van Puijenbroek, L. Bosquee, A. P. Meert, D. Schallier, J. C. Goeminne, G. Tits, P. Collard, K. Nackaerts, J. L. Canon, F. Duplaquet, D. Galdermans, P. Germonpre, M. A. Azerad, G. Vandenhoven, J. De Greve, and J. Vansteenkiste. Gefitinib monotherapy in advanced nonsmall cell lung cancer: a large western community implementation study. *Eur Respir J*, 29(1):128–33, 2007.

36. R. A. Walgren, M. A. Meucci, and H. L. McLeod. Pharmacogenomic discovery approaches: will the real genes please stand up? *J Clin Oncol*, 23(29):7342–9, 2005.

37. J. P. Bonjour, T. Chevalley, R. Rizzoli, and S. Ferrari. Gene-environment interactions in the skeletal response to nutrition and exercise during growth. *Med Sport Sci*, 51:64–80, 2007.

38. F. Ma, T. Sun, Y. Shi, D. Yu, W. Tan, M. Yang, C. Wu, D. Chu, Y. Sun, B. Xu, and D. Lin. Polymorphisms of egfr predict clinical outcome in advanced non-small-cell lung cancer patients treated with gefitinib. *Lung Cancer*, 66(1):114–9, 2009.

39. L. Zhang, X. Yuan, Y. Chen, X. J. Du, S. Yu, and M. Yang. Role of egfr snps in survival of advanced lung adenocarcinoma patients treated with gefitinib. *Gene*, 517(1):60–4, 2013.

40. W. al Saleh, S. L. Giannini, N. Jacobs, M. Moutschen, J. Doyen, J. Boniver, and P. Delvenne. Correlation of t-helper secretory differentiation and types of antigen-presenting cells in squamous intraepithelial lesions of the uterine cervix. *J Pathol*, 184(3):283–90, 1998.

41. T. D. de Gruijl, H. J. Bontkes, J. M. Walboomers, M. J. Stukart, F. S. Doekhie, A. J. Remmink, T. J. Helmerhorst, R. H. Verheijen, M. F. Duggan-Keen, P. L. Stern, C. J. Meijer, and R. J. Scheper. Differential t helper cell responses to human papillomavirus type 16 e7 related to viral clearance or persistence in patients with cervical neoplasia: a longitudinal study. *Cancer Res*, 58(8):1700–6, 1998.

42. S. K. Hussain, M. M. Madeleine, L. G. Johnson, Q. Du, M. Malkki, H. W. Wilkerson, F. M. Farin, J. J. Carter, D. A. Galloway, J. R. Daling, E. W. Petersdorf, and S. M. Schwartz. Cervical and vulvar cancer risk in relation to the joint effects of cigarette smoking and genetic variation in interleukin 2. *Cancer Epidemiol Biomarkers Prev*, 17(7):1790–9, 2008.

43. K. L. Fung, J. Pan, S. Ohnuma, P. E. Lund, J. N. Pixley, C. Kimchi-Sarfaty, S. V. Ambudkar, and M. M. Gottesman. Mdr1 synonymous polymorphisms alter transporter specificity and protein stability in a stable epithelial monolayer. *Cancer Res*, 74(2):598–608, 2014.

44. Wen-Feng Yu, Agneta Nordberg, Rivka Ravid, and Zhi-Zhong Guan. Correlation of oxidative stress and the loss of the nicotinic receptor alpha4 subunit in the temporal cortex of patients with alzheimer's disease. *Neuroscience Letters*, 338(1):13 – 16, 2003.

45. P. M. Greenwood, M. K. Lin, R. Sundararajan, K. J. Fryxell, and R. Parasuraman. Synergistic effects of genetic variation in nicotinic and muscarinic receptors on visual attention but not working memory. *Proc Natl Acad Sci U S A*, 106(9):3633–8, 2009.

46. I. Reinvang, A. J. Lundervold, H. Rootwelt, E. Wehling, and T. Espeseth. Individual variation in a cholinergic receptor gene modulates attention. *Neurosci Lett*, 453(3):131–4, 2009.

47. S. Markett, C. Montag, and M. Reuter. The nicotinic acetylcholine receptor gene chrna4 is associated with negative emotionality. *Emotion*, 11(2):450–5, 2011.

48. S. J. Tsai, H. L. Yeh, C. J. Hong, Y. J. Liou, A. C. Yang, M. E. Liu, and J. P. Hwang. Association of chrna4 polymorphism with depression and loneliness in elderly males. *Genes Brain Behav*, 11(2):230–4, 2012.

49. R. H. Swerdlow and S. J. Kish. Mitochondria in alzheimer's disease. *Int Rev Neurobiol*, 53:341–85, 2002.

50. Z. Syed, F. Dudbridge, and L. Kent. An investigation of the neurotrophic factor genes gdnf, ngf, and nt3 in susceptibility to adhd. *Am J Med Genet B Neuropsychiatr Genet*, 144B(3):375–8, 2007.

51. X. Xu, J. Mill, K. Zhou, K. Brookes, C. K. Chen, and P. Asherson. Family-based association study between brain-derived neurotrophic factor gene polymorphisms and attention deficit hyperactivity disorder in uk and taiwanese samples. *Am J Med Genet B Neuropsychiatr Genet*, 144B(1):83–6, 2007.

52. A. C. Conner, C. Kissling, E. Hodges, R. Hunnerkopf, R. M. Clement, E. Dudley, C. M. Freitag, M. Rosler, W. Retz, and J. Thome. Neurotrophic factor-related gene polymorphisms and adult attention deficit hyperactivity disorder (adhd) score in a high-risk male population. *Am J Med Genet B Neuropsychiatr Genet*, 147B(8):1476–80, 2008.

53. M. Ribases, A. Hervas, J. A. Ramos-Quiroga, R. Bosch, A. Bielsa, X. Gastaminza, M. Fernandez-Anguiano, M. Nogueira, N. Gomez-Barros, S. Valero, M. Gratacos, X. Estivill, M. Casas, B. Cormand, and M. Bayes. Association study of 10 genes encoding neurotrophic factors and their receptors in adult and child attention-deficit/hyperactivity disorder. *Biol Psychiatry*, 63(10):935–45, 2008.

54. S. Park, B. N. Kim, J. W. Kim, M. S. Shin, S. C. Cho, J. H. Kim, J. W. Son, Y. M. Shin, U. S. Chung, and D. H. Han. Neurotrophin 3 genotype and emotional adverse effects of osmotic-release oral system methylphenidate (oros-mph) in children with attention-deficit/hyperactivity disorder. *J Psychopharmacol*, 28(3):220–6, 2014.

55. D. Blum, R. Hourez, M. C. Galas, P. Popoli, and S. N. Schiffmann. Adenosine receptors and huntington's disease: implications for pathogenesis and therapeutics. *Lancet Neurol*, 2(6):366–74, 2003.

56. P. Popoli, D. Blum, A. Martire, C. Ledent, S. Ceruti, and M. P. Abbracchio. Functions, dysfunctions and possible therapeutic relevance of adenosine a2a receptors in huntington's disease. *Prog Neurobiol*, 81(5-6):331–48, 2007.

57. P. Popoli, D. Blum, M. R. Domenici, S. Burnouf, and Y. Chern. A critical evaluation of adenosine a2a receptors as potentially "druggable" targets in huntington's disease. *Curr Pharm Des*, 14(15):1500–11, 2008.

58. C. M. Dhaenens, S. Burnouf, C. Simonin, E. Van Brussel, A. Duhamel, L. Defebvre, C. Duru, I. Vuillaume, C. Cazeneuve, P. Charles, P. Maison, S. Debruxelles, C. Verny, H. Gervais, J. P. Azulay, C. Tranchant, A. C. Bachoud-Levi, A. Durr, L. Buee, P. Krystkowiak, B. Sablonniere, and D. Blum. A genetic variation in the adora2a gene modifies age at onset in huntington's disease. *Neurobiol Dis*, 35(3):474–6, 2009.

59. M. Vital, E. Bidegain, V. Raggio, and P. Esperon. Molecular characterization of genes modifying the age at onset in huntington's disease in uruguayan patients. *Int J Neurosci*, pages 1–17, 2015.

60. P. Seeman. Dopamine receptors and the dopamine hypothesis of schizophrenia. *Synapse*, 1(2):133–52, 1987.

61. S. Gupta, S. Jain, S. K. Brahmachari, and R. Kukreti. Pharmacogenomics: a path to predictive medicine for schizophrenia. *Pharmacogenomics*, 7(1):31–47, 2006.

62. M. Gupta, C. Chauhan, P. Bhatnagar, S. Gupta, S. Grover, P. K. Singh, M. Purushottam, O. Mukherjee, S. Jain, S. K. Brahmachari, and R. Kukreti. Genetic susceptibility to schizophrenia: role of dopaminergic pathway gene polymorphisms. *Pharmacogenomics*, 10(2):277–91, 2009.

63. C. Virgos, L. Martorell, J. Valero, L. Figuera, F. Civeira, J. Joven, A. Labad, and E. Vilella. Association study of schizophrenia with polymorphisms at six candidate genes. *Schizophr Res*, 49(1-2):65–71, 2001.

64. C. Dubertret, L. Gouya, N. Hanoun, J. C. Deybach, J. Ades, M. Hamon, and P. Gorwood. The 3' region of the drd2 gene is involved in genetic susceptibility to schizophrenia. *Schizophr Res*, 67(1):75–85, 2004.

65. S. J. Glatt and E. G. Jonsson. The cys allele of the drd2 ser311cys polymorphism has a dominant effect on risk for schizophrenia: evidence from fixed- and random-effects meta-analyses. *Am J Med Genet B Neuropsychiatr Genet*, 141B(2):149–54, 2006.

66. B. R. Lawford, R. M. Young, C. D. Swagell, M. Barnes, S. C. Burton, W. K. Ward, K. R. Heslop, S. Shadforth, A. van Daal, and C. P. Morris. The c/c genotype of the c957t polymorphism of the dopamine d2 receptor is associated with schizophrenia. *Schizophr Res*, 73(1):31–7, 2005.

67. K. Hanninen, H. Katila, O. Kampman, S. Anttila, A. Illi, R. Rontu, K. M. Mattila, J. Hietala, M. Hurme, E. Leinonen, and T. Lehtimaki. Association between the c957t polymorphism of the dopamine d2 receptor gene and schizophrenia. *Neurosci Lett*, 407(3):195–8, 2006.

68. J. Hoenicka, M. Aragues, R. Rodriguez-Jimenez, G. Ponce, I. Martinez, G. Rubio, M. A. Jimenez-Arriero, and T. Palomo. C957t drd2 polymorphism is associated with schizophrenia in spanish patients. *Acta Psychiatr Scand*, 114(6):435–8, 2006.

69. M. Monakhov, V. Golimbet, L. Abramova, V. Kaleda, and V. Karpov. Association study of three polymorphisms in the dopamine d2 receptor gene and schizophrenia in the russian population. *Schizophr Res*, 100(1-3):302–7, 2008.

70. E. T. Betcheva, T. Mushiroda, A. Takahashi, M. Kubo, S. K. Karachanak, I. T. Zaharieva, R. V. Vazharova, II Dimova, V. K. Milanova, T. Tolev, G. Kirov, M. J. Owen, M. C. O'Donovan, N. Kamatani, Y. Nakamura, and D. I. Toncheva. Case-control association study of 59 candidate genes reveals the drd2 snp rs6277 (c957t) as the only susceptibility factor for schizophrenia in the bulgarian population. *J Hum Genet*, 54(2):98–107, 2009.

71. J. Duan, M. S. Wainwright, J. M. Comeron, N. Saitou, A. R. Sanders, J. Gelernter, and P. V. Gejman. Synonymous mutations in the human dopamine receptor d2 (drd2) affect mrna stability and synthesis of the receptor. *Hum Mol Genet*, 12(3):205–16, 2003.

72. H. Asaga, K. Akiyama, T. Ohsawa, and A. Ishigami. Increased and type ii-specific expression of peptidylarginine deiminase in activated microglia but not hyperplastic astrocytes following kainic acid-evoked neurodegeneration in the rat brain. *Neurosci Lett*, 326(2):129–32, 2002.

73. M. M. Ahmed, H. Hoshino, T. Chikuma, M. Yamada, and T. Kato. Effect of memantine on the levels of glial cells, neuropeptides, and peptide-degrading enzymes in rat brain regions of ibotenic acid-treated alzheimer's disease model. *Neuroscience*, 126(3):639–49, 2004.

74. Y. Watanabe, A. Nunokawa, N. Kaneko, T. Arinami, H. Ujike, T. Inada, N. Iwata, H. Kunugi, M. Itokawa, T. Otowa, N. Ozaki, and T. Someya. A two-stage case-control association study of padi2 with schizophrenia. *J Hum Genet*, 54(7):430–2, 2009.

75. K. Stenius, R. Janz, T. C. Sudhof, and R. Jahn. Structure of synaptogyrin (p29) defines novel synaptic vesicle protein. *J Cell Biol*, 131(6 Pt 2):1801–9, 1995.

76. R. Janz, T. C. Sudhof, R. E. Hammer, V. Unni, S. A. Siegelbaum, and V. Y. Bolshakov. Essential roles in synaptic plasticity for synaptogyrin i and synaptophysin i. *Neuron*, 24(3):687–700, 1999.

77. K. Mirnics, F. A. Middleton, A. Marquez, D. A. Lewis, and P. Levitt. Molecular characterization of schizophrenia viewed by microarray analysis of gene expression in prefrontal cortex. *Neuron*, 28(1):53–67, 2000.

78. R. Verma, C. Chauhan, Q. Saleem, C. Gandhi, S. Jain, and S. K. Brahmachari. A nonsense mutation in the synaptogyrin 1 gene in a family with schizophrenia. *Biol Psychiatry*, 55(2):196–9, 2004.

79. R. Verma, S. Kubendran, S. K. Das, S. Jain, and S. K. Brahmachari. Syngr1 is associated with schizophrenia and bipolar disorder in southern india. *J Hum Genet*, 50(12):635–40, 2005.

80. Luda Diatchenko, Gary D. Slade, Andrea G. Nackley, Konakporn Bhalang, Asgeir Sigurdsson, Inna Belfer, David Goldman, Ke Xu, Svetlana A. Shabalina, Dmitry Shagin, Mitchell B. Max, Sergei S. Makarov, and William Maixner. Genetic basis for individual variations in pain perception and the development of a chronic pain condition. *Human Molecular Genetics*, 14(1):135–143, 2005.

81. Trude Teoline Rakvåg, Pål Klepstad, Cecilie Baar, Tor Morten Kvam, Ola Dale, Stein Kaasa, Hans Einar Krokan, and Frank Skorpen. The Val158Met polymorphism of the human catechol-O-methyltransferase (COMT) gene may influence morphine requirements in cancer pain patients. *Pain*, 116(1-2):73–78, 2005.

82. Emmanuelle Martinez, Françoise Silvy, Lombardo Dominique, and Mas Eric. Single nucleotide polymorphisms and risk factors predictive of pancreatic adenocarcinoma. *Cancer Cell & Microenvironment*, 3:e1231, mar 2016.

83. J. C. Cohen, R. S. Kiss, A. Pertsemlidis, Y. L. Marcel, R. McPherson, and H. H. Hobbs. Multiple rare alleles contribute to low plasma levels of hdl cholesterol. *Science*, 305(5685):869–72, 2004.

84. S. Romeo, L. A. Pennacchio, Y. Fu, E. Boerwinkle, A. Tybjaerg-Hansen, H. H. Hobbs, and J. C. Cohen. Population-based resequencing of angptl4 uncovers variations that reduce triglycerides and increase hdl. *Nat Genet*, 39(4):513–6, 2007.

85. S. Kathiresan, C. J. Willer, G. M. Peloso, S. Demissie, K. Musunuru, E. E. Schadt, L. Kaplan, D. Bennett, Y. Li, T. Tanaka, B. F. Voight, L. L. Bonnycastle, A. U. Jackson, G. Crawford, A. Surti, C. Guiducci, N. P. Burtt, S. Parish, R. Clarke, D. Zelenika, K. A. Kubalanza, M. A. Morken, L. J. Scott, H. M. Stringham, P. Galan, A. J. Swift, J. Kuusisto, R. N. Bergman, J. Sundvall, M. Laakso, L. Ferrucci, P. Scheet, S. Sanna, M. Uda, Q. Yang, K. L. Lunetta, J. Dupuis, P. I. de Bakker, C. J. O'Donnell, J. C. Chambers, J. S. Kooner, S. Hercberg, P. Meneton, E. G. Lakatta, A. Scuteri, D. Schlessinger, J. Tuomilehto, F. S. Collins, L. Groop, D. Altshuler, R. Collins, G. M. Lathrop, O. Melander, V. Salomaa, L. Peltonen, M. Orho-Melander, J. M. Ordovas, M. Boehnke, G. R. Abecasis, K. L. Mohlke, and L. A. Cupples. Common variants at 30 loci contribute to polygenic dyslipidemia. *Nat Genet*, 41(1):56–65, 2009.

86. T. M. Teslovich, K. Musunuru, A. V. Smith, A. C. Edmondson, I. M. Stylianou, M. Koseki, J. P. Pirruccello, S. Ripatti, D. I. Chasman, C. J. Willer, C. T. Johansen, S. W. Fouchier, A. Isaacs, G. M. Peloso, M. Barbalic, S. L. Ricketts, J. C. Bis, Y. S. Aulchenko, G. Thorleifsson, M. F. Feitosa, J. Chambers, M. Orho-Melander, O. Melander, T. Johnson, X. Li, X. Guo, M. Li, Y. Shin Cho, M. Jin Go, Y. Jin Kim, J. Y. Lee, T. Park, K. Kim, X. Sim, R. Twee-Hee Ong, D. C. Croteau-Chonka, L. A. Lange, J. D. Smith, K. Song, J. Hua Zhao, X. Yuan, J. Luan, C. Lamina, A. Ziegler,

W. Zhang, R. Y. Zee, A. F. Wright, J. C. Witteman, J. F. Wilson, G. Willemsen, H. E. Wichmann, J. B. Whitfield, D. M. Waterworth, N. J. Wareham, G. Waeber, P. Vollenweider, B. F. Voight, V. Vitart, A. G. Uitterlinden, M. Uda, J. Tuomilehto, J. R. Thompson, T. Tanaka, I. Surakka, H. M. Stringham, T. D. Spector, N. Soranzo, J. H. Smit, J. Sinisalo, K. Silander, E. J. Sijbrands, A. Scuteri, J. Scott, D. Schlessinger, S. Sanna, V. Salomaa, J. Saharinen, C. Sabatti, A. Ruokonen, I. Rudan, L. M. Rose, R. Roberts, M. Rieder, B. M. Psaty, P. P. Pramstaller, I. Pichler, M. Perola, B. W. Penninx, N. L. Pedersen, C. Pattaro, A. N. Parker, G. Pare, B. A. Oostra, C. J. O'Donnell, M. S. Nieminen, D. A. Nickerson, G. W. Montgomery, T. Meitinger, R. McPherson, M. I. McCarthy, et al. Biological, clinical and population relevance of 95 loci for blood lipids. *Nature*, 466(7307):707–13, 2010.

87. D. Evans, J. Arzer, J. Aberle, and F. U. Beil. Rare variants in the lipoprotein lipase (lpl) gene are common in hypertriglyceridemia but rare in type iii hyperlipidemia. *Atherosclerosis*, 214(2):386–90, 2011.

88. M. Wada, H. Marusawa, R. Yamada, A. Nasu, Y. Osaki, M. Kudo, M. Nabeshima, Y. Fukuda, T. Chiba, and F. Matsuda. Association of genetic polymorphisms with interferon-induced haematologic adverse effects in chronic hepatitis c patients. *J Viral Hepat*, 16(6):388–96, 2009.

89. C. Vidal, A. Cachia, and A. Xuereb-Anastasi. Effects of a synonymous variant in exon 9 of the cd44 gene on pre-mrna splicing in a family with osteoporosis. *Bone*, 45(4):736–42, 2009.
